# ALKBH5 modulates hematopoietic stem and progenitor cell energy metabolism through m^6^A modification-mediated RNA stability control

**DOI:** 10.1016/j.celrep.2023.113163

**Published:** 2023-09-23

**Authors:** Yimeng Gao, Joshua T. Zimmer, Radovan Vasic, Chengyang Liu, Rana Gbyli, Shu-Jian Zheng, Amisha Patel, Wei Liu, Zhihong Qi, Yaping Li, Raman Nelakanti, Yuanbin Song, Giulia Biancon, Andrew Z. Xiao, Sarah Slavoff, Richard G. Kibbey, Richard A. Flavell, Matthew D. Simon, Toma Tebaldi, Hua-Bing Li, Stephanie Halene

**Affiliations:** 1 Section of Hematology, Department of Internal Medicine, Yale Cancer Center, and Yale Center for RNA Science and Medicine, Yale University School of Medicine, New Haven, CT 06520, USA; 2 Yale Stem Cell Center, Yale University School of Medicine, New Haven, CT 06520, USA; 3 Department of Molecular Biophysics & Biochemistry, Yale University, New Haven, CT 06511, USA; 4 Institute for Biomolecular Design and Discovery, Yale University, West Haven, CT 06516, USA; 5 Department of Medicine, University of Toronto, Toronto, ON M5S3H2, Canada; 6 Department of Genetics and Yale Stem Cell Center, Yale School of Medicine, New Haven, CT 06520, USA; 7 Department of Chemistry, Yale University, New Haven, CT 06520, USA; 8 Department of Hematologic Oncology, Sun Yat-sen University Cancer Center, State Key Laboratory of Oncology in South China, Collaborative Innovation Center for Cancer Medicine, Guangzhou 510060, China; 9 Department of Internal Medicine, Yale University, New Haven, CT 06520, USA; 10 Department of Cellular & Molecular Physiology, Yale University, New Haven, CT 06520, USA; 11 Department of Immunobiology, Yale University School of Medicine, New Haven, CT 06520, USA; 12 Howard Hughes Medical Institute, Chevy Chase, MD 20815, USA; 13 Department of Cellular, Computational and Integrative Biology (CIBIO), University of Trento, 38123 Trento, Italy; 14 Shanghai Institute of Immunology, State Key Laboratory of Oncogenes and Related Genes, Shanghai Jiao Tong University School of Medicine, Shanghai 200025, China; 15 Department of Pathology, Yale University School of Medicine, New Haven, CT 06520, USA

**Keywords:** m^6^A modification, RNA stability, ALKBH5, hematopoietic stem and progenitor cells, ATP production, energy metabolism, oxidative phosphorylation, OXPHOS, stress hematopoiesis, leukemia

## Abstract

*N*^6^-methyladenosine (m^6^A) RNA modification controls numerous cellular processes. To what extent these post-transcriptional regulatory mechanisms play a role in hematopoiesis has not been fully elucidated. We here show that the m^6^A demethylase alkB homolog 5 (ALKBH5) controls mitochondrial ATP production and modulates hematopoietic stem and progenitor cell (HSPC) fitness in an m^6^A-dependent manner. Loss of ALKBH5 results in increased RNA methylation and instability of oxoglutarate-dehydrogenase (*Ogdh*) messenger RNA and reduction of OGDH protein levels. Limited OGDH availability slows the tricarboxylic acid (TCA) cycle with accumulation of α-ketoglutarate (α-KG) and conversion of α-KG into L-2-hydroxyglutarate (L-2-HG). L-2-HG inhibits energy production in both murine and human hematopoietic cells *in vitro*. Impaired mitochondrial energy production confers competitive disadvantage to HSPCs and limits clonogenicity of *Mll-AF9*-induced leukemia. Our study uncovers a mechanism whereby the RNA m^6^A demethylase ALKBH5 regulates the stability of metabolic enzyme transcripts, thereby controlling energy metabolism in hematopoiesis and leukemia.

## Highlights

• Loss of ALKBH5 attenuates hematopoietic fitness in response to stress
• ALKBH5 regulates the stability of *Ogdh* mRNA and of other metabolic enzymes
• L-2-HG accumulates in response to OGDH downregulation and attenuates the energy metabolism
• L-2-HG inhibits proliferation of both normal and malignant hematopoietic cells

## Graphical abstract

**Figure:**
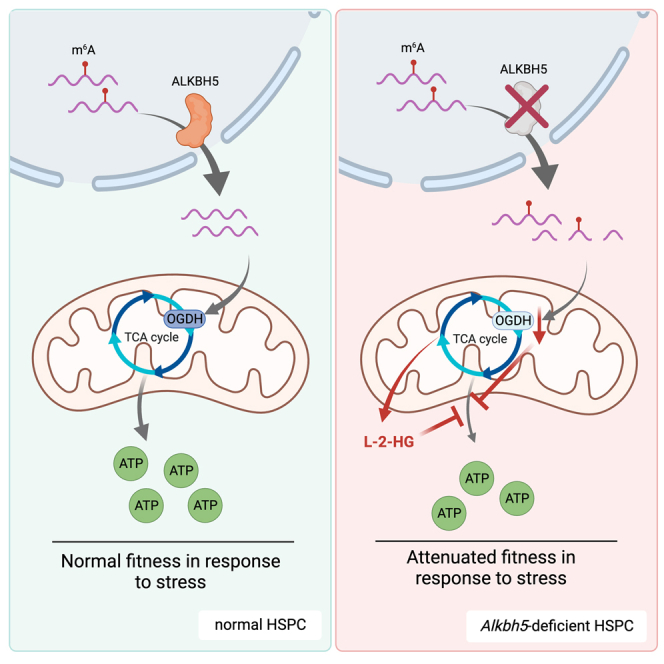

## Teaser

Gao et al. find that ALKBH5 regulates hematopoietic stem and progenitor cell fitness in response to stress. ALKBH5 modulates energy metabolism by controlling stability of *Ogdh* and other metabolic enzyme transcripts. Accumulation of L-2-HG further inhibits the energy metabolism of hematopoietic cells. These findings provide insights on ALKBH5 in hematopoiesis.

## Introduction

The mRNA modification *N*^6^-methyladenosine (m^6^A) regulates numerous cellular processes through modulation of RNA stability and translation efficiency.^1^^,^^2^^,^^3^ Recent comprehensive reviews provide an overview of advances in the m^6^A RNA field.^4^^,^^5^^,^^6^ The core component of the m^6^A writer complex, METTL3, has been shown to be essential for hematopoietic development by our group and others.^7^^,^^8^^,^^9^^,^^10^^,^^11^^,^^12^ m^6^A readers have distinct and overlapping functions in regulation of RNA stability and translational efficiency. The m^6^A reader YTHDF2 regulates hematopoietic stem cell (HSC) regeneration in an mRNA-dependent manner.^13^^,^^14^ YTHDF3 regulates HSC function by targeting the m^6^A modifications on *Ccnd1*, *Foxm1*, and *Axl1*.^15^^,^^16^ YTHDC1 has been shown to be essential for DNA replication during leukemogenesis.^17^ The m^6^A reader IGF2BP2 regulates HSC function through modulating stability of *Bmi1*.^18^ Among the erasers, the role of the m^6^A and m^6^A_m_ demethylase fat mass and obesity-associated protein (FTO) has been extensively studied.^19^^,^^20^^,^^21^ FTO has been shown to contribute to oncogenesis in acute myeloid leukemia (AML)^22^ and R-2-hydroxyglutarate (R-2-HG), a metabolite produced by mutant IDH, inhibits FTO function, limiting aerobic glycolysis in AML.^23^ Less is known about the m^6^A demethylase alkB homolog 5 (ALKBH5) and its role in hematopoiesis. *Alkbh5* KO mice are viable and carry a mild defect in spermatogenesis.^24^ Suppression of ALKBH5 in virally infected cells inhibits viral replication by limiting the availability of tricarboxylic acid (TCA) cycle intermediates, in particular itaconate, which is essential for viral replication.^25^ Recently, two groups found that loss of ALKBH5 in AML results in reduced levels of *TACC3* and *AXL1*, respectively, limiting AML survival. Comparatively, ALKBH5 seemed dispensable during steady-state hematopoiesis.^26^^,^^27^ Given the interest in targeting m^6^A RNA erasers in AML, detailed studies of normal and stress hematopoiesis are necessary to fully understand the implications of disrupting this pathway.

Hematopoiesis is tightly regulated. The TCA cycle and mitochondrial respiration provide ATP and essential metabolites to meet the high demands during hematopoietic cell proliferation, differentiation, and maturation.^28^ Energy metabolism switches from glycolysis in long-term HSCs (LT-HSCs) to mitochondrial respiration in short-term HSCs (ST-HSCs) and committed progenitor cells.^29^ The mechanism by which HSCs control the temporal switch of their metabolic machinery is not fully understood.

Changes in mRNA stability serve as a rapid and tight control mechanism of transcript availability and translation, and its regulation by m^6^A RNA post-transcriptional modification represents an attractive mechanism that could play a role in temporal regulation of the metabolic switch in the hematopoietic hierarchy.^30^^,^^31^ We here use a hematopoiesis-specific *Alkbh5*-deficient murine system and model loss of ALKBH5 in *Mll-AF9*-driven leukemia to show that ALKBH5 regulates the hematopoietic stem and progenitor cell metabolic switch from glycolysis to oxidative phosphorylation (OXPHOS) via control of stability of metabolic gene transcripts, in particular of the TCA rate-limiting enzyme *Ogdh*.

## Results

### Loss of ALKBH5 limits competitive HSPC repopulation and proliferation but not steady-state hematopoiesis

**Figure 1:**
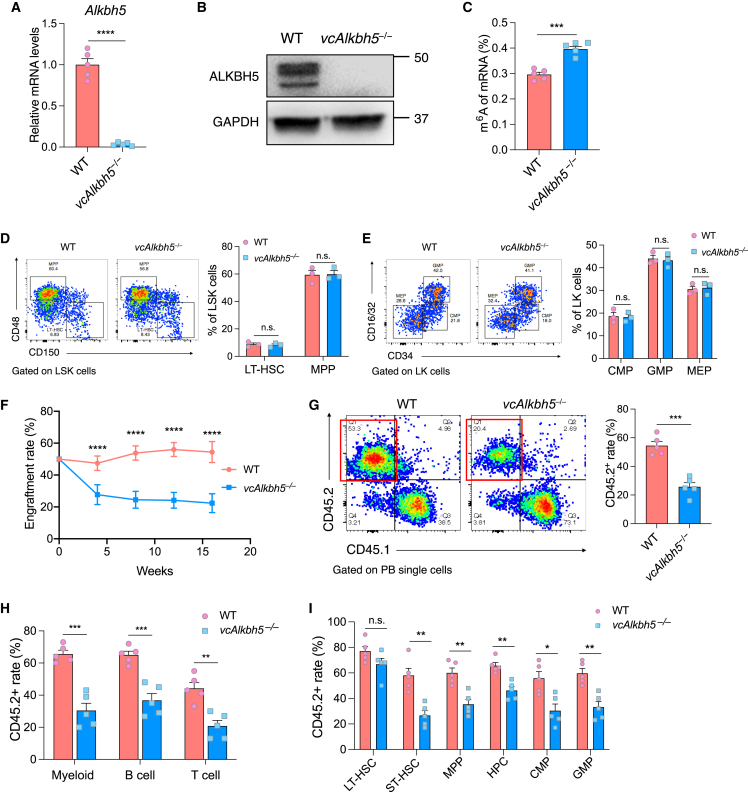
ALKBH5 is dispensable for steady-state hematopoiesis but required for competitive repopulation (A) Measurement of *Alkbh5* mRNA expression levels in mouse bone marrow (BM) by qRT-PCR (n = 5 of each group). (B) Measurement of ALKBH5 protein levels in mouse BM by immunoblot. (C) Quantification of RNA m^6^A modification in mouse BM by ELISA (n = 5 of each group). (D) Gating strategy and quantification of long-term hematopoietic stem cell (LT-HSC) and multipotent progenitor (MPP) frequencies by flow cytometry (n = 3 of each group). (E) Gating strategy and quantification of granulocyte-monocyte progenitors (GMPs), common myeloid progenitors (CMPs), and megakaryocyte-erythroid progenitors (MEPs) in BM (n = 3 of each group). (F) Competitive transplantation assay measuring CD45.2^+^ donor-derived cells in the peripheral blood (PB) of recipient mice 4, 8, 12, and 16 weeks after BM transplantation (n = 5 recipients of each group). (G) Gating strategy and quantification of PB engraftment of CD45.2^+^ donor cells, highlighted by red quadrants, and WT CD45.1^+^ competitor cells by flow cytometry, at 16 weeks post-transplantation (n = 5 recipients of each group). (H) Contribution of donor cells to myeloid and lymphoid lineages in PB as determined by flow cytometry (n = 5 recipients of each group). Myeloid, CD11b^+^; B cells, B220^+^; T cells, CD3^+^. (I) Contribution of CD45.2^+^ donor cells to the HSC and progenitor compartments in the BM as determined by flow cytometry (n = 5 recipients of each group). LT-HSC, Lin^–^Sca-1^+^c-Kit^+^CD150^+^CD48^–^; ST-HSC (short-term HSC), Lin^–^Sca-1^+^c-Kit^+^CD150^–^CD48^–^; MPP, Lin^–^Sca-1^+^c-Kit^+^CD150^–^CD48^+^; HPC (hematopoietic progenitor cell), Lin^–^Sca-1^+^c-Kit^+^CD150^+^CD48^+^; CMP, Lin^–^Sca-1^–^c-Kit^+^CD34^+^CD16/32^–^; GMP, Lin^–^Sca-1^–^c-Kit^+^CD34^+^CD16/32^+^. Data are represented as mean ± SEM and are representative of at least three independent experiments; p values were calculated using two-tailed Student’s t test. n.s., not significant, ^∗^p < 0.05, ^∗∗^p < 0.01, ^∗∗∗^p < 0.001, ^∗∗∗∗^p < 0.0001.

To dissect the role of ALKBH5 in hematopoietic stem and progenitor cell function, we generated *Vav*-iCre; *Alkbh5*^fl/fl^ mice, which specifically delete *Alkbh5* in the hematopoietic lineage at embryonic day 11.5 (E11.5).^32^^,^^33^ *Alkbh5* mRNA and protein were efficiently depleted in bone marrow (BM) of *Vav*-iCre^+^; *Alkbh5*^fl/fl^ (*vcAlkbh5*^−/−^) mice, with a concomitant increase in m^6^A RNA modification compared with *Vav*-iCre^–^; *Alkbh5*^fl/fl^ (wild-type [WT]) BM (Figures 1A–1C).

*vcAlkbh5*^−/−^ mice were viable, and their hematopoietic parameters are comparable to those of WT mice (Figure S1A). Hematopoietic stem and progenitor cell (HSPC) relative frequency, absolute number, and differentiation potential were unperturbed (Figures 1D, 1E, S1B, and S1C). These results are in keeping with previous studies that found ALKBH5 to be dispensable for normal hematopoiesis.^26^^,^^27^

To determine whether ALKBH5 plays a role in HSPC fitness in response to stress, we tested the hematopoietic reconstitution potential of *Alkbh5*-deficient HSPCs in competitive transplantation assays. CD45.2^+^ WT or *vcAlkbh5*^−/−^ BM cells were transplanted in equal numbers with congenic CD45.1^+^ *Pep3b* marrow into lethally irradiated *Pep3b* recipient mice. Engraftment rates 16 weeks after transplantation showed a significant reduction of *vcAlkbh5*^−/−^ CD45.2^+^ cells compared with WT CD45.2^+^ cells (Figures 1F and 1G). Hematopoietic reconstitution in peripheral blood was deficient across all lineages, including the myeloid, B, and T cell compartments (Figure 1H). Assessment of lineage distribution within the CD45.2^+^ peripheral blood (PB) cells showed a statistically significant relative reduction in myeloid cells in *vcAlkbh5*^−/−^ compared with WT CD45.2^+^ PB cells over time, without significant differences in B and T cell contributions (Figure S1D). At the designated endpoint of 16 weeks post-transplantation, we dissected the BM HSPC compartment. *vcAlkbh5*^−/−^ CD45.2^+^ cells exhibited significantly reduced contribution to all HSPC subpopulations, except for phenotypic LT-HSCs (Lin^–^Sca-1^+^c-Kit^+^CD150^+^CD48^–^), which were preserved (Figure 1I). To further assay HSC function, we performed secondary transplantations and found that *vcAlkbh5*^−/−^ CD45.2^+^ cells performed worse than in primary transplantation, now also revealing a competitive defect at the LT-HSC stage (Figures S1E and S1F).

To determine whether the competitive repopulation disadvantage was attributable to a homing defect, we injected carboxyfluoroscein succinimidyl ester (CFSE)-labeled BM cells from WT or *vcAlkbh5*^−/−^ mice into lethally irradiated CD45.1^+^ recipient mice followed by quantification of CFSE^+^ cells in the BM and spleen of recipients 16 h after transplantation (Figure S2A). Fewer *vcAlkbh5*^−/−^ cells homed to the recipient BM and spleen compared with WT cells, suggesting that decreased homing efficiency of *vcAlkbh5*^−/−^ BM cells may contribute to the decreased competitive advantage of *vcAlkbh5*^−/−^ cells (Figures S2B and S2C).

To determine whether the observed competitive disadvantage in transplantation assays was exclusively attributable to homing defects or to cell-extrinsic factors in primary *vcAlkbh5*^−/−^ mice, we generated Cre^+^ and Cre^–^ reverse tetracycline-transactivator (rtTA)-*Alkbh5*^fl/fl^ mice, which allow for induction of *Alkbh5* deletion by treating recipient mice with doxycycline (Dox) after transplantation (Figure S2D). We competitively transplanted Cre^+^ rtTA-*Alkbh5*^fl/fl^ (hereafter named *CTA5*^−/−^) and Cre^–^ rtTA-*Alkbh5*^fl/fl^ (hereafter named CTA5^fl/fl^) BM cells at a 1:1 ratio with WT CD45.1^+^ competitor BM into lethally irradiated *Pep3b* recipients and confirmed comparable engraftment rates in all mice prior to Dox treatment (Figure S2E). Following Dox induction, PB contribution by *CTA5*^−/−^ cells declined gradually and was significantly lower compared with CTA5^fl/fl^ cells by 16 weeks after deletion of *Alkbh5* (Figure S2F). Declining PB chimerism was accompanied by significantly lower CD45.2^+^ *CTA5*^−/−^ cells in recipient BM 16 weeks after Dox treatment, suggesting that *Alkbh5*^−/−^ HSPCs carry a cell-intrinsic defect under replicative stress (Figures S2G and S2H). To determine whether *Alkbh5*^−/−^ HSPCs would exhibit impaired recovery after challenge with 5-fluorouracil, we treated *Alkbh5*^−/−^ and WT mice with 5-fluorouracil (5-FU). Recovery post-5-FU was similar in knockout (KO) and WT mice in PB and among stem and progenitor cell compartments (Figures S1G and S1H). Together, these data suggest that loss of ALKBH5 affects homing efficiency as well as cellular proliferation capacity during hematopoietic reconstitution in competitive transplantation.

**Figure 2:**
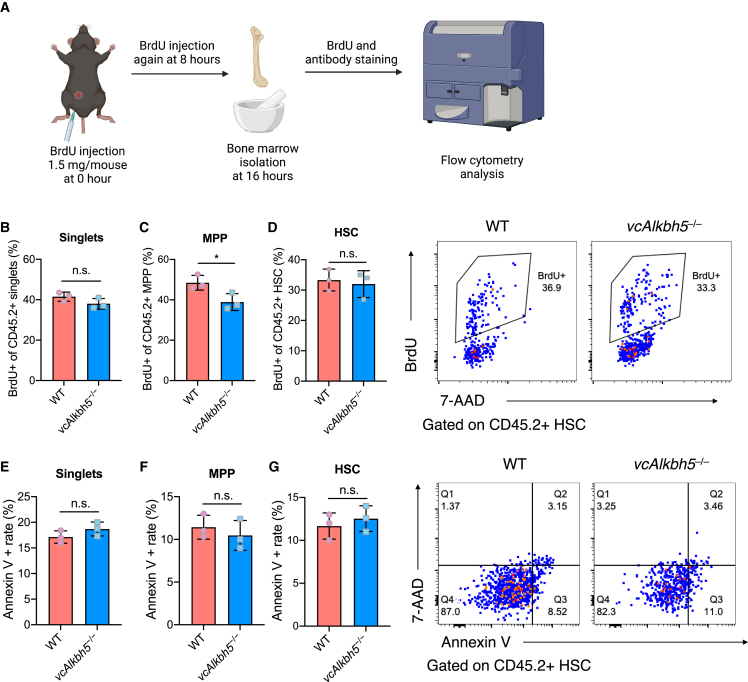
Loss of ALKBH5 attenuates hematopoietic stem and progenitor cell proliferation without causing apoptosis in transplanted mice (A) Schematic diagram of assessment of cell proliferation by BrdU administration in competitively transplanted mice. (B–D) Quantification of CD45.2^+^ singlet (B), MPP (C), and HSC (D) proliferation via BrdU uptake and 7-AAD staining of DNA content by flow cytometry (n = 3 of each group). (E–G) Determination and quantification of apoptotic rate of CD45.2^+^ singlets (E), MPPs (F), and HSCs (G) via Annexin V staining (n = 3 of each group). Data are represented as mean ± SEM and are representative of at least three independent experiments; p values were calculated using two-tailed Student’s t test. n.s., not significant, ^∗^p < 0.05.

To further characterize cellular function driving this competitive disadvantage, we measured proliferation and apoptosis of WT and *vcAlkbh5*^−/−^ BM subpopulations in the competitively transplanted mice (Figure 2A). *In vivo* uptake of bromodeoxyuridine (BrdU) suggested a proliferation defect in *vcAlkbh5*^−/−^ whole BM nucleated cells and in LT-HSCs but was not statistically significant (Figures 2B and 2D). CD45.2^+^ *vcAlkbh5*^−/−^ multipotent progenitors (MPPs) showed a significant decrease in proliferation compared with WT MPPs in competitively transplanted mice (Figure 2C). Annexin V staining revealed no difference in *vcAlkbh5*^−/−^ whole BM, MPP, or LT-HSC apoptotic rates (Figures 2E–2G). These results suggest that loss of ALKBH5 affects proliferation but does not induce apoptosis in HSPCs under replication stress.

### Loss of ALKBH5 destabilizes the *Ogdh* transcript in a m^6^A modification-dependent manner

**Figure 3:**
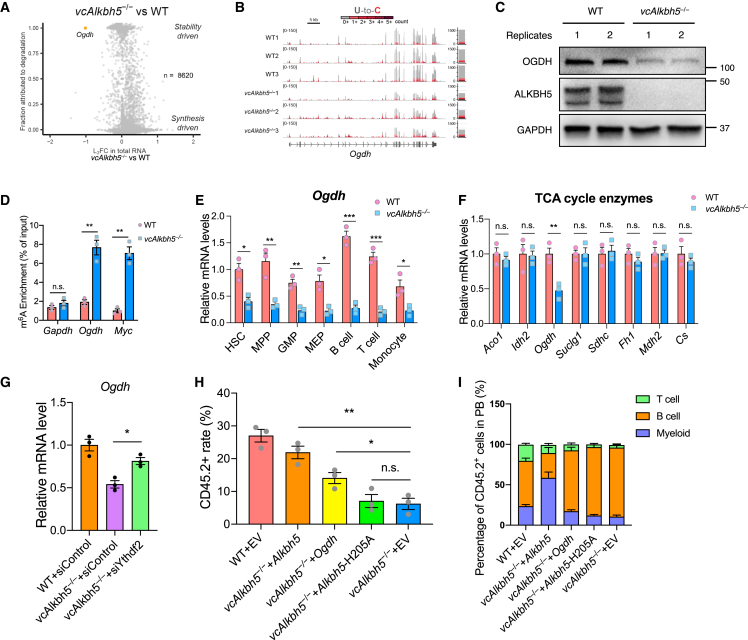
Loss of ALKBH5 results in reduced OGDH mRNA and protein level, and restoration of ODGH expression rescues competitive reconstitution (A) Scatterplot of gene expression fold change and the relative contribution of RNA degradation of *vcAlkbh5*^−/−^ vs. WT lineage-depleted BM cells (n = 3 of each group). (B) TimeLapse-seq tracks depicting the read coverage over *Ogdh* for WT and *vcAlkbh5*^−/−^ groups. Bar graphs of normalized read counts aligned to the mature *Ogdh* transcript are shown at the right. Reads are colored according to their U-to-C mutational content. (C) Measurement of OGDH protein level in WT and *vcAlkbh5*^−/−^ BM cells. (D) Measurement of m^6^A enrichment of *Ogdh* mRNA in BM cells by m^6^A-RIP-qPCR (n = 3 of each group). Results are presented relative to input. *Gapdh* serves as negative control, and *Myc* serve as positive control. (E) *Ogdh* mRNA levels in different cell types of WT and *vcAlkbh5*^−/−^ BM cells, normalized to *Actb* (n = 3 of each group). (F) Determination of gene expression levels of TCA cycle enzymes by qRT-PCR, normalized to *Actb* (n = 3 of each group). (G) *Ogdh* expression level upon knockdown of *Ythdf2* as measured by qPCR. (H) Engraftment rate of *vcAlkbh5*^−/−^ lineage-depleted BM cells transduced with empty or *Alkbh5*-, *Ogdh*-, or *Alkbh5*-H205A-expressing retroviral vector, 16 weeks after transplantation (n = 3 of each group). (I) Characterization of multilineage population of rescued cells in the recipient mice (n = 3 of each group). Data are represented as mean ± SEM and are representative of at least three independent experiments; the p values were calculated using two-tailed Student’s t test. ^∗^p < 0.05, ^∗∗^p < 0.01, ^∗∗∗^p < 0.001.

We next sought to understand the molecular mechanisms underlying the hematopoietic reconstitution defects of *vcAlkbh5*^−/−^ HSPCs in the competitive transplantation challenge. It has been shown that m^6^A modification destabilizes target mRNAs through increased RNA degradation.^34^^,^^35^ To identify targets that may underly the HPSC defect and to dissect whether changes in stability or transcription underly expression changes, we applied TimeLapse-seq, which labels newly transcribed transcripts via 4-thiouridine (s^4^U) incorporation, to sequence lineage-depleted BM cells of WT and *vcAlkbh5*^−/−^ mice.^36^ s^4^U labeling distinguishes newly transcribed RNAs from pre-existing transcripts via introduction of U-to-C mutations at the time of reverse transcription, yielding insights into mRNA turnover rates. As evident in prior studies, loss of ALKBH5 resulted in few altered transcripts.^25^^,^^37^ We only identified *Ddi2* and *Rps4l* as significantly upregulated in *vcAlkbh5*^−/−^ lineage-depleted BM cells. Given that m^6^A RNA modification specifically affects RNA stability, we analyzed RNA turnover in the TimeLapse-seq data and identified 77 mRNAs that were significantly destabilized in *vcAlkbh5*^−/−^ compared with WT cells, with significant enrichment for metabolic processes within these 77 genes (Figures S3A–S3C). Among the destabilized transcripts, *Ogdh* was the most significantly downregulated, and its reduction was entirely attributable to RNA decay (Figure 3A) without changes in synthesis rates (Figure 3B). Stabilized transcripts were few and were not enriched for a particular pathway. The transcript stability and turnover rates of house-keeping genes, shown for *Actb*, and key hematopoietic transcription factors, shown for *Cebpa*, remained unchanged (Figure S3D). Immunoblot confirmed reduction of OGDH protein levels in BM of *vcAlkbh5*^−/−^ mice (Figure 3C). m^6^A RNA modification of the *Ogdh* transcript was significantly increased in the BM of *vcAlkbh5*^−/−^ mice as determined by m^6^A RNA immunoprecipitation (RIP)-PCR, while no differences were detected in control genes, such as *Gapdh* (Figure 3D). Although m^6^A modification was also increased on the *Myc* transcript, its protein levels remained unchanged, suggesting that OGDH and MYC are differentially affected by loss of ALKBH5, despite ALKBH5-regulated m^6^A modification of their transcripts (Figure S3D). These results suggest that ALKBH5 directly regulates *Ogdh* RNA stability and protein levels through m^6^A RNA modification.

To determine at which differentiation stage in the hematopoietic hierarchy loss of ALKBH5 led to reduced *Ogdh* RNA stability, we measured *Ogdh* mRNA levels in sorted BM hematopoietic subpopulations via qRT-PCR. *Ogdh* RNA was significantly decreased in all HSPC subpopulations and mature cellular compartments (Figure 3E). *Ogdh* is the only TCA enzyme within the 77 destabilized transcripts. To confirm these findings, we measured mRNA expression levels of key metabolic enzymes of the TCA cycle and found only *Ogdh* to be significantly reduced in the lineage-depleted *vcAlkbh5*^−/−^ BM cells (Figure 3F). A previous study suggested that YTHDF2 is the predominant reader that mediates the effects of ALKBH5-mediated m^6^A erasure.^25^^,^^27^ We therefore transfected *vcAlkbh5*^−/−^ lineage-depleted BM cells with *Ythdf2* or control small interfering RNA (siRNA) and confirmed that knockdown of *Ythdf2* could increase *Ogdh* mRNA levels (Figure 3G). Next, to confirm that downregulation of OGDH was the underlying cause for the competitive disadvantage of *Alkbh5*-deficient cells, we overexpressed ALKBH5 or OGDH in lineage-depleted *vcAlkbh5*^−/−^ BM cells and assessed their functional rescue in competitive transplantation assays. ALKBH5 re-expression restored the engraftment efficiency of *vcAlkbh5*^−/−^ donor cells, whereas re-expression of the catalytically dead H205A mutant ALKBH5 had no effect (Figure 3H). Re-expression of ALKBH5 specifically rescued myeloid lineage reconstitution, the lineage that was most affected by loss of ALKBH5 (Figure 3I). OGDH overexpression also significantly increased the engraftment rate of *vcAlkbh5*^−/−^ donor cells, albeit less efficiently than ALKBH5 rescue (Figure 3H). Re-expression of ALKBH5 and OGDH in *vcAlkbh5*^−/−^ lineage-depleted cells also rescued the homing defect (Figure S3F). These results confirm a critical role for ALKBH5 in hematopoietic response to proliferative stress, mediated in part by the TCA cycle intermediate OGDH.

### Loss of ALKBH5 affects TCA cycle and modulates energy production of HSPCs

**Figure 4:**
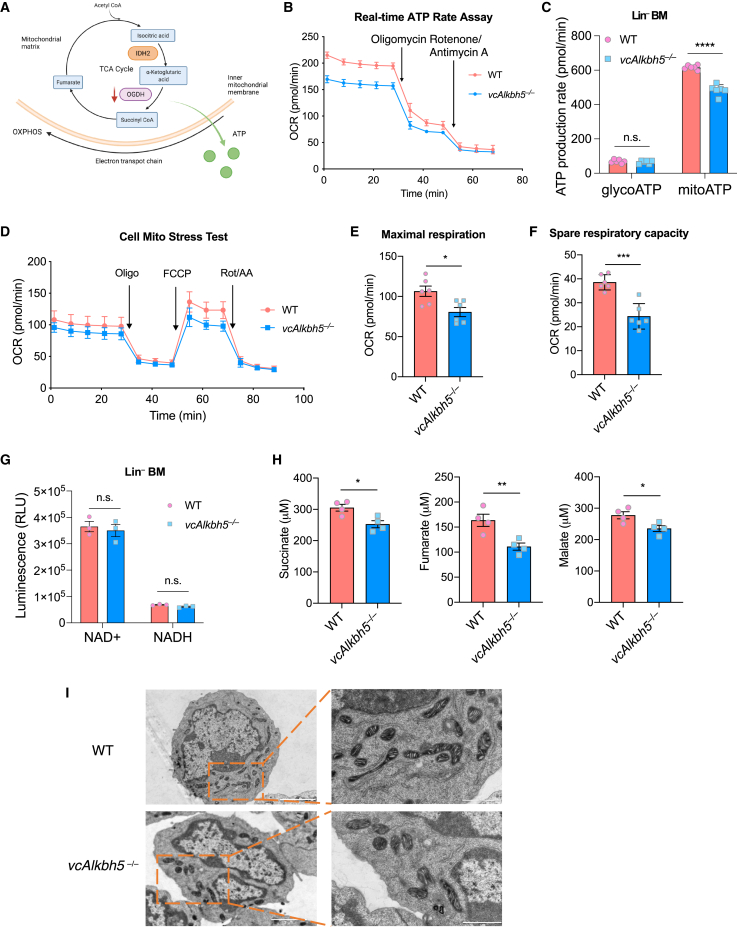
*vcAlkbh5*^−/−^ hematopoietic cells are defective in mitochondrial energy production (A) Diagram depicting expected consequences of reduced OGDH levels on energy production. (B) Real-time analysis of oxygen consumption rates (OCRs) of WT and *vcAlkbh5*^−/−^ lineage-depleted BM cells via the Seahorse XF ATP Rate Assay (n = 6 of each group). (C) Quantification of ATP production rate via glycolysis versus mitochondrial respiration, determined via Seahorse ATP Rate Assay (n = 6 of each group). (D) Determination of mitochondrial respiration function via measurement of the OCR using the Cell Mito Stress Assay in WT and *vcAlkbh5*^−/−^ lineage-depleted BM cells. (E and F) Quantification of the maximal respiration rate (E) and spare respiratory capacity (F) determined by the Seahorse XF Cell Mito Stress Assay (n = 6 of each group). (G) Determination of NAD^+^ and NADH levels in the WT and *vcAlkbh5*^−/−^ lineage-depleted BM cells (n = 3 of each group). (H) Quantification of metabolite levels of TCA cycle in murine plasma via ELISA (n = 4 of each group). (I) Ultrastructure of mitochondria in lineage-depleted BM cells of WT and *vcAlkbh5*^−/−^ mice imaged via electron microscopy. Scale bar, 2 μm (left) and 1 μm (right). Data are represented as mean ± SEM and are representative of at least two independent experiments; p values were calculated using two-tailed Student’s t test. n.s., not significant, ^∗^p < 0.05, ^∗∗^p < 0.01, ^∗∗∗^p < 0.001, ^∗∗∗∗^p < 0.0001.

OGDH is a rate-limiting enzyme in the TCA cycle, converting α-ketoglutarate (α-KG) to succinyl CoA. The TCA cycle is intricately linked to mitochondrial OXPHOS for adenosine triphosphate (ATP) production (Figure 4A). We therefore sought to understand whether reduced levels of OGDH in *vcAlkbh5*^−/−^ BM cells would affect energy metabolism in vc*Alkbh5*^−/−^ hematopoiesis. We applied the Seahorse ATP Rate Assay^38^ to lineage-depleted BM cells from *vcAlkbh5*^−/−^ and WT mice to measure the oxygen consumption rate (OCR), which reflects the mitochondrial respiration of cells. *vcAlkbh5*^−/−^ lineage-depleted cells showed lower basal OCRs compared with WT cells, as measured by sequential inhibition of OXPHOS with oligomycin (Oligo) and rotenone/antimycin A (Rot/AA) (Figure 4B). Based on the OCR and on H^+^ production, mitochondrial ATP (mitoATP) production was significantly lower in *vcAlkbh5*^−/−^ than in WT cells, while ATP production from glycolysis (glycoATP) did not differ between the two groups (Figure 4C).

To confirm that reduced OGDH levels compromise OXPHOS kinetics, we further applied the Seahorse XF Cell Mito Stress Test to specifically measure mitochondrial activity in lineage-depleted BM cells. *vcAlkbh5*^−/−^ and WT cells responded with similar OCR kinetics to inhibitors of OXPHOS. However, as evident in the Seahorse ATP Rate Assay, basal and peak OCRs were reduced in *vcAlkbh5*^−/−^ cells (Figure 4D), revealing a significantly lower maximal respiration and spare respiratory capacity in *vcAlkbh5*^−/−^ compared with WT cells (Figures 4E and 4F).

NAD^+^ serves as an important co-factor in the OGDH-catalyzed reaction of converting α-KG to succinyl CoA, and its reduction could also affect energy production via OXPHOS. Therefore, we measured NAD^+^ and NADH levels in lineage-depleted HSPCs and found no difference between the two groups (Figures 4G and S4A). However, quantitation of metabolites downstream of OGDH in the TCA cycle revealed significant reduction of succinate, fumarate, and malate in the plasma of *vcAlkbh5*^−/−^ mice, indicating reduced TCA cycle capacity as a result of reduction of the rate-limiting enzyme OGDH (Figure 4H). Measurements of glycolytic capacity in WT and *vcAlkbh5*^−/−^ HSPCs by the specific Seahorse Glycolysis Stress Test showed no difference in glycolysis and glycolytic capacity between *vcAlkbh5*^−/−^ and WT cells (Figure S4B). Furthermore, in the competitively transplanted mice, the CD45.2^+^ cells derived from *vcAlkbh5*^−/−^ BM also demonstrated the ATP production defects at similar levels as observed in primary *vcAlkbh5*^−/−^ mic, suggesting that they are sufficient to result in a competitive disadvantage (Figure S4C).

To determine whether reduced OXPHOS was affected at the mitochondrial membrane, we measured mitochondrial membrane potentials via the MitoProbe JC-1 assay, revealing no difference between *vcAlkbh5*^−/−^ and WT cells (Figure S4D). MitoTracker Green staining of BM HSC and MPP populations also revealed no changes in mitochondrial mass between *vcAlkbh5*^−/−^ and WT cells (Figure S4E). Similarly, mitochondrial ultrastructure and number were also intact in *vcAlkbh5*^−/−^ cells as determined by electron microscopy (EM) (Figures 4I and S4F). Together, these results suggest that loss of ALKBH5 compromises mitochondrial energy production via perturbation of the TCA cycle without disrupting mitochondrial ultrastructure.

### Increased L-2-HG inhibits energy production of normal and malignant hematopoietic cells

**Figure 5:**
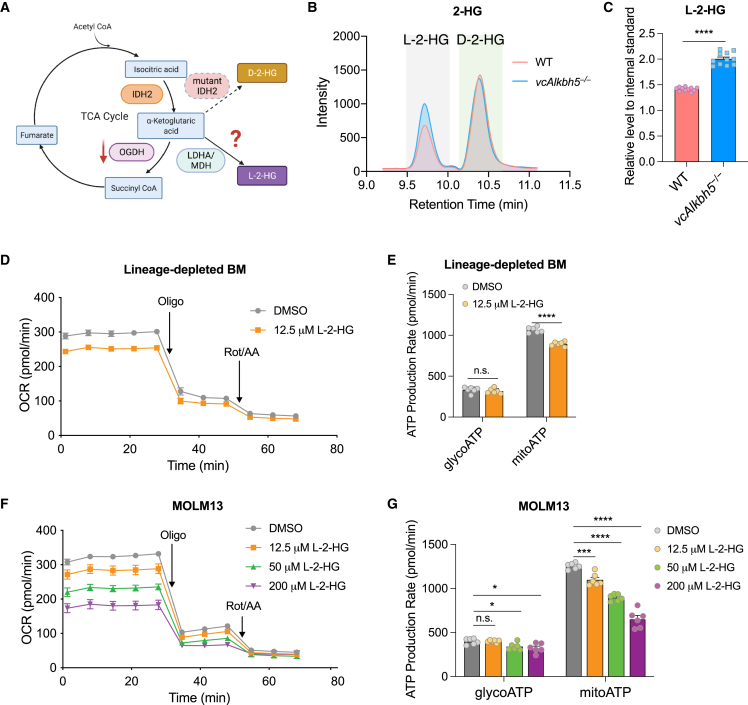
Reduced OGDH results in accumulation of L-2-HG that compromises mitochondrial respiration (A) Diagram depicting expected consequences of reduced OGDH levels on TCA cycle metabolites. (B) Chiral liquid chromatography-mass spectrometry (LC-MS) analysis resolving enantiomers of L-2-HG and D-2-HG in plasma of WT and *vcAlkbh5*^−/−^ mice. Shaded areas serve as reference marking L- (gray) and D-enantiomer (mint) retention times. (C) Quantification of L-2-HG levels in plasma of WT and *vcAlkbh5*^−/−^ mice (n = 13 biological independent samples of each group). (D) Real-time analysis of the OCR of murine WT lineage-depleted BM cells treated with vehicle control or L-2-HG. (E) Quantification of the ATP production rate via glycolysis versus mitochondrial respiration in murine lineage-depleted BM cells treated with vehicle control or L-2-HG (n = 6 of each group). (F) Real-time analysis of the OCR of MOLM13 cells treated with vehicle control and increasing concentrations of L-2-HG. (G) Quantification of the ATP production rate via glycolysis versus mitochondrial respiration in MOLM13 cells treated with vehicle control or increasing concentrations of L-2-HG as determined by the Seahorse ATP Rate Assay (n = 6 of each group). Oligo, oligomycin; Rot/AA, rotenone and antimycin A. Data are represented as mean ± SEM and are representative of at least two independent experiments; the p values were calculated using two-tailed Student’s t test. n.s., not significant, ^∗^p < 0.05, ^∗∗∗^p < 0.001, ^∗∗∗∗^p < 0.0001.

Reduction of OGDH has been shown to result in accumulation of α-KG.^39^ As a result, α-KG can be converted to L-2-HG via induction of lactate dehydrogenase (LDH) and malate dehydrogenase (MDH) activity.^40^ This typically occurs in response to hypoxia to offset the adverse consequences of mitochondrial reductive stress.^41^ The oncometabolite and enantiomer of L-2-HG, D-2-HG, is produced in cancer cells with isocitrate dehydrogenase (IDH) 1 and 2 mutations, including AML,^42^ with profound effects on hematopoiesis.^43^ L- and D-2-HG have similar physiological effects and are usually measured in parallel.^44^ To determine whether loss of ALKBH5 and reduction of OGDH result in increased levels of L- or D-2-HG, we measured L- and D-2-HG in plasma of WT and *vcAlkbh5*^−/−^ mice by chiral derivatization, which distinguishes these two enantiomers (Figure 5A).^45^ While D-2-HG levels remained unaffected, L-2-HG levels were significantly increased in plasma of *vcAlkbh5*^−/−^ compared with WT mice (Figures 5B and 5C), concurrent with a small but significant increase in α-KG levels (Figures S5A and S5B).

Previous studies have demonstrated that both D- and L-2-HG can inhibit various α-KG-dependent dioxygenases, including JmjC-KDM and TET enzymes.^39^^,^^46^^,^^47^^,^^48^ In addition, L-2-HG was found to be increased under hypoxia conditions.^41^^,^^47^ Therefore, L-2-HG could mediate metabolic adaptation and ATP production under hypoxic conditions. This could also explain the close relationship between ALKBH5 and cancer progression under hypoxic conditions reported by other groups.^49^^,^^50^

To test whether L-2-HG has a direct inhibitory effect on ATP production, we treated murine WT lineage-depleted BM cells with L-2-HG at physiological concentrations and determined cellular OCR and ATP production via the Seahorse ATP Rate Assay.^44^^,^^51^ Murine lineage-depleted BM cells treated with L-2-HG displayed attenuated OCR kinetics, and while their glycoATP production was unchanged, their mitoATP production was significantly reduced compared with vehicle-treated cells (Figures 5D and 5E). To determine whether L-2-HG had similar effects in human cells, we treated human MOLM13 cells with increasing, physiologically achievable concentrations of L-2-HG. MOLM13 cells showed significantly reduced mitoATP production in response to L-2-HG in a dose-dependent manner (Figures 5F and 5G). Only at very high, supra-physiological concentrations (100 μM) did L-2-HG exhibit a direct inhibitory effect on cell proliferation without resulting in increased apoptosis (Figures S5C and S5D). These results demonstrate that human and murine hematopoietic cells respond to increased L-2-HG levels via reduced oxygen consumption and ATP production, highlighting the versatile mechanisms by which ALKBH5 can regulate cellular metabolism.

### ALKBH5 depletion disrupts AML progression through m^6^A-mediated energy metabolism control

**Figure 6:**
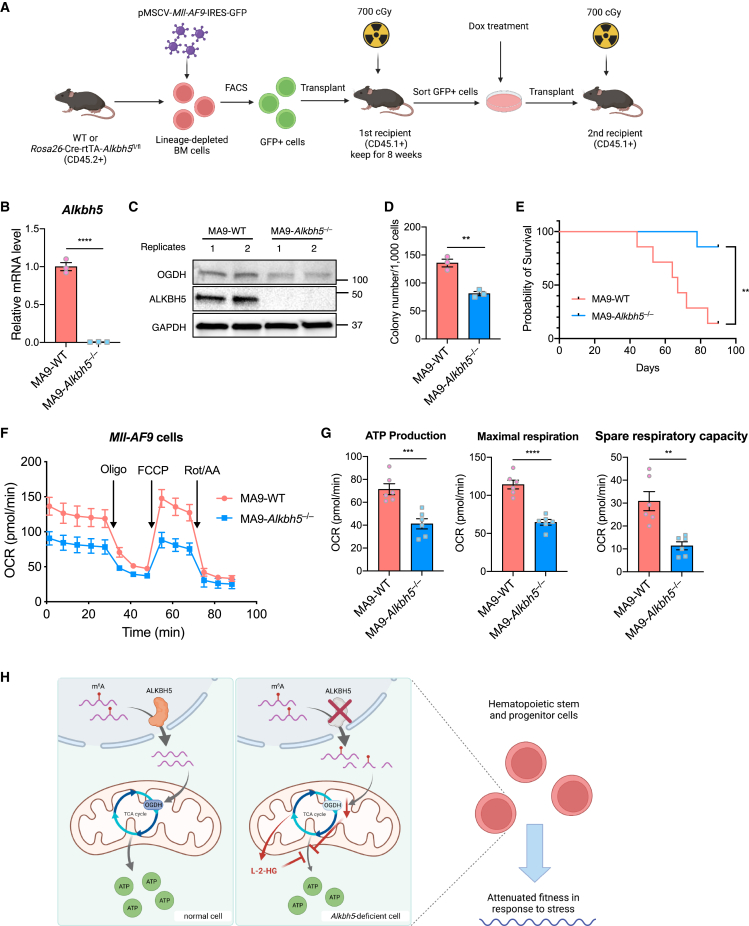
Loss of ALKBH5 limits *Mll-AF9*-induced leukemogenicity via diminished energy metabolism (A) Diagram depicting the establishment of *Mll-AF9* acute myeloid leukemia (AML). (B) Determination of deletion of *Alkbh5* mRNA level in *Mll-AF9* BM cells by qRT-PCR after treatment of Dox for 4 days (n = 3 of each group). (C) Measurement of OGDH and ALKBH5 protein levels in the *Mll-AF9* AML cell lines. (D) Assessment of colony-forming unit (CFU) potential of MA9-WT and MA9-*Alkbh5*^−/−^ AML cells (n = 3 of each group). (E) Kaplan-Meier survival curves for recipient mice of MA9-WT and MA9-*Alkbh5*^−/−^ AML cells (n = 7 recipients of each group). (F) Determination of mitochondrial respiration function via measurement of the OCR using the Cell Mito Stress Assay in *Mll-AF9* cells (n = 6 of each group). (G) Quantification of the ATP production, maximal respiration, and spare respiratory capacity by the Seahorse XF Cell Mito Stress Assay (n = 6 of each group). (H) Schematic depicting the role of ALKBH5 and its downstream effect on hematopoietic cell function. Oligo, oligomycin; Rot/AA, rotenone and antimycin A. Data are represented as mean ± SEM and are representative of at least two independent experiments; the p values of (B), (D), and (G) were calculated using two-tailed Student’s t test. The p value of (E) was calculated using log-rank test. ^∗∗^p < 0.01, ^∗∗∗^p < 0.001, ^∗∗∗∗^p < 0.0001.

Previous studies have shown a critical role for ALKBH5 in myeloid leukemia.^26^^,^^27^ To determine whether ALKBH5-mediated regulation of energy metabolism is required for leukemogenesis in addition to reduction of *TACC3* and *AXL1* to limit AML survival, we generated *Mll-AF9* mutant leukemia from Cre-rtTA-*Alkbh5*^fl/fl^ mice.^52^ Lineage-depleted BM cells from WT and Cre-rtTA-*Alkbh5*^fl/fl^ mice were transduced with pMSCV-*Mll-AF9*-IRES-GFP retrovirus, and GFP^+^ cells were transplanted into sublethally irradiated recipient mice (Figure 6A). WT and inducible KO Mll-AF9 cell lines (MA9-WT and MA9-*Alkbh5*^fl/fl^) were propagated in mice or in culture in Dox-free medium. Dox treatment for 4 days efficiently deleted *Alkbh5* at the mRNA and protein levels in the MA9-*Alkbh5*^−/−^ cell line (Figures 6B and 6C).

We next tested proliferation rate *in vitro* by colony-forming unit (CFU) assay. MA9-*Alkbh5*^−/−^ cells proliferated significantly slower than MA9-WT cells, and MA9-*Alkbh5*^−/−^ colonies were smaller than MA9-WT colonies (Figures 6D and S6A). We also characterized the leukemic potential of MA9-WT and MA9-*Alkbh5*^−/−^ cells by transplantation into sublethally irradiated recipients and measured engraftment and survival rates. MA9-*Alkbh5*^−/−^ cells showed significantly reduced engraftment with less severe splenomegaly and enhanced survival of recipient mice (Figures 6E, S6B, and S6C), concordant with previous reports.^26^^,^^27^ To confirm the cause of death for MA9-WT recipients, we analyzed BM and spleens of recipient mice and confirmed involvement by leukemia; in recipients of MA9-*Alkbh5*^−/−^ cells leukemia was also present, but normal splenic architecture was at least partially preserved (Figure S6D). MA9-*Alkbh5*^−/−^ cells did not show increased apoptotic rates compared with MA9-WT cells (Figure S6E), suggesting instead a proliferative defect in MA9-*Alkbh5*^−/−^ leukemia. To confirm that the ALKBH5-OGDH axis at least in part limits leukemogenesis in Mll-AF9 leukemia, we transfected MA9-WT cells with *Ogdh* or control siRNA. *Ogdh* knockdown significantly limited CFU formation compared with control siRNA (Figure S6F). To further determine whether compromised energy metabolism could underlie this phenotype, we comprehensively assayed MA9-WT and MA9-*Alkbh5*^−/−^ cells in the Cell Mito Stress Assay. The Cell Mito Stress Assay confirmed that MA9-*Alkbh5*^−/−^ cells displayed significantly attenuated energy production as measured by real-time OCR (Figure 6F), and MA9-*Alkbh5*^−/−^ cells showed significantly reduced ATP production and maximal respiration and spare respiratory capacity compared with MA9-WT cells (Figure 6G).

These results suggest that loss of ALKBH5 compromises *Mll-AF9*-mediated leukemogenicity at least in part through attenuated energy production caused by reduced OGDH levels, as also seen in *Alkbh5*-deficient HSPCs.

## Discussion

Mice with constitutional deletion of *Alkbh5* are viable but demonstrate a defect in spermatogenesis.^24^^,^^53^ Since then, ALKBH5 has been shown to regulate diverse cellular functions. ALKBH5 regulates the effect of immunotherapy via modulating lactate levels and suppressive immune cell accumulation in the tumor microenvironment.^54^ Loss of ALKBH5 inhibits viral replication by downregulation of host cell itaconate levels,^25^ and ALKBH5 has also been shown to be essential for tissue development and cellular function in the immune system and pancreatic tissues.^55^^,^^56^ We have previously shown the importance of m^6^A RNA modification via deletion of its writer *Mettl3* in the highly complex and temporally regulated hematopoietic system.^7^ We here show that, similarly, the m^6^A eraser ALKBH5 is critical to hematopoietic function.

In this study, we used the *Vav*-iCre mouse model to specifically delete *Alkbh5* in the hematopoietic system and the Dox-inducible Cre-rtTa-*Alkbh5*^fl/fl^ mouse model to allow temporal regulation of *Alkbh5* deletion. These models allow us to study ALKBH5 function without the use of pI:pC, a double-strand RNA that induces significant inflammation and the interferon-responsive *Mx1* promoter. Unlike in *Mx1-Cre*-driven or constitutive CRISPR-Cas9-mediated *Alkbh5*-deleted mouse models, which displayed unchanged or even increased engraftment advantage during competitive transplantation, respectively,^26^^,^^27^ we uncover a significant competitive transplantation disadvantage of HSPCs with both *Vav*-iCre and Dox-inducible Cre-mediated *Alkbh5* deletion. Unlike in Vav-iCre and Dox-inducible Cre mice, in constitutively (CRISPR-Cas9) *Alkbh5*^−/−^ mice, HSPCs reside in an *Alkbh5*^−/−^ BM microenvironment, potentially affecting HSPC repopulation ability. We employ the innovative TimeLapse-seq technology to identify transcripts whose expression is up- or downregulated with the specific advantage of distinguishing transcriptional from RNA stability regulatory mechanisms. Only a small number of transcripts are differentially regulated in *Alkbh5*^−/−^ cells in addition to *Alkbh5* itself, and *Ogdh* is the most significantly downregulated transcript, entirely explained by increased m^6^A modification and transcript instability. Dysregulation of a limited number of transcripts has previously been shown for loss of ALKBH5,^25^ distinguishing it from FTO’s broader effects that demethylate m^6^A and 2′-*O*-methyladenosine (m^6^A_m_).^5^

Loss of ALKBH5 significantly reduces mRNA stability and protein levels of the rate-limiting TCA cycle enzyme OGDH, compromising OXPHOS and ATP production. In addition, reduced conversion of α-KG to succinyl CoA results in increased L-2-HG levels that in turn compromise mitochondrial energy production (Figure 6H). The effects of D-2-HG have been extensively studied in the setting of leukemia because of its accumulation in patients with IDH1/2 mutations.^42^^,^^57^ As the enantiomer of D-2-HG, L-2-HG follows similar mechanisms as D-2-HG, but its role in hematopoietic development and pathologic conditions has not been thoroughly studied.

It is possible that dysregulation of the metabolic switch from glycolysis in LT-HSCs to OXPHOS in ST-HSCs^58^^,^^59^ contributes to the competitive disadvantage we observe in *Alkbh5*-deficient cells in competitive transplantation assays, while steady-state hematopoiesis remains unaffected in *Alkbh5*^−/−^ mice. As would be expected, phenotypic *Alkbh5*^−/−^ LT-HSCs are mostly spared in competitively transplanted mice, suggesting that their reliance on glycolysis rather than OXPHOS for energy production preserves their proliferative capacity.^29^^,^^60^^,^^61^ ST-HSCs and more mature progeny that rely on OXPHOS, on the other hand, are significantly reduced. The reliance on glycolysis in LT-HSCs with a switch to OXPHOS in ST-HSCs and beyond serves to protect LT stem cells from possible damage caused by excessive reactive oxygen species.^62^^,^^63^^,^^64^ Unfortunately, the Seahorse assay’s requirements preclude analysis of limited-number populations. We therefore cannot exclude that more primitive HSC populations also exhibit altered energy metabolism. Our findings are in keeping with recent studies that have shown that cell metabolism pathways, including amino acid catabolism and protein synthesis, critically maintain normal function and fitness of HSCs under stress and aging conditions.^65^^,^^66^

Unlike FTO, which has demethylase activities on both m^6^A and m^6^A_m_, ALKBH5 has limited effects on normal tissue development and function at steady state. Recent studies have focused on the role of ALKBH5 in the context of tumorigenesis^67^ and viral infection.^25^ Our studies showcase the importance of ALKBH5 at the level of stem cell regulation. As in viral infection, ALKBH5-mediated regulation of OGDH levels regulate TCA cycle activity and impact cellular metabolic fitness. *Alkbh5-*deleted mice are viable, but male mice are infertile due to compromised spermatogenesis, the mechanism of which is only partially understood.^53^ It is possible that loss of ALKBH5-mediated regulation of metabolism underlies disruption of this highly energy-dependent process also.

Previous studies have shown that *ALKBH5* knockdown activates apoptosis and the p53 pathway while also significantly suppressing E2F and MYC targets, G2M checkpoint, and mitotic spindle pathways. Shen et al. further dissect the role of TACC3 in AML as the most significant target of ALKBH5, as its expression levels correlated with prognosis in human AML.^26^ Wang et al. identified that knockdown of ALKBH5 resulted in reduced expression of genes related to amino acid metabolism, cell cycle, and PI3K/AKT signaling in primary human AML, while genes associated with apoptosis and differentiation were upregulated.^27^ We similarly derived a “metabolic signature” from genes destabilized in *Alkbh5*^−/−^ non-leukemic BM cells. The magnitude of effect of disruption of these pathways is likely to differ between healthy and leukemic cells.

Combination of ALKBH5 inhibition with drugs targeting other metabolic pathways could be leveraged in the treatment of leukemia.^68^^,^^69^^,^^70^^,^^71^ On the other hand, the knowledge that inhibition of ALKBH5 may compromise HSPC function will also be critical to understanding and mitigating on-target hematopoietic toxicities. In our MA9 leukemia mouse model, loss of ALKBH5 does not fully eliminate leukemia cells but does slow the progression of leukemia. The target of ALKBH5, OGDH, has been shown to inhibit the proliferation of glioblastoma and gastric cancer cells.^72^^,^^73^ Therefore, the study of ALKBH5 regulation in hematopoiesis could provide potential targets for leukemia treatment in the future.

In summary, we here demonstrate that ALKBH5 controls metabolic switch and energy production in HSPCs by regulating the stability of metabolic enzyme transcripts through its m^6^A demethylase activity. Our results highlight the complexity and critical role of m^6^A RNA modification as a regulator of energy metabolism during hematopoietic development and leukemogenesis.

### Limitations of the study

The most significant physiological defect of ALKBH5 deficiency exists in competitive transplantation, and we have shown the rescue effect after overexpression of ALKBH5 and OGDH. However, it would be very informative if we could also measure the restoration of energy metabolism *in vivo*. At present, restoration of competitive engraftment will have to suffice as a functional surrogate test. We also sought to find additional functional readouts of the defects in *vcAlkbh5*^−/−^ hematopoiesis in addition to competitive transplantation. 5-FU treatment failed to elicit a difference between WT and *vcAlkbh5*^−/−^ mice, possibly due to the short nature of the challenge or due to the lack of competing WT cells that serve to uncover the metabolic defect of *vcAlkbh5*^−/−^ HSPCs. Previous work has shown that downregulation of *Tacc3* or *Axl1* upon deletion of *Alkbh5* limits leukemogenesis. We here show that *Alkbh5*-deficient *Mll*-*AF9* leukemia cells are also affected by the defects in the energy metabolism pathway. Future studies may be necessary to dissect the relative contributions of OGDH and previously reported pathways, especially as leukemia treatments targeting the m^6^A epitranscriptome are being developed.

## STAR★Methods

### Key resources table

**Table:** 

REAGENT or RESOURCE	SOURCE	IDENTIFIER
**Antibodies**		

Purified Rat Anti-Mouse CD16/CD32 (Mouse BD Fc Block™)	BD Biosciences	Cat# 553142; RRID: AB_394657
Miltenyi lineage detection Cocktail-Biotin, mouse	Miltenyi	Cat# 130-092-613; RRID: AB_1103214
APC-Cy7 Streptavidin	Biolegend	Cat# 405208
PE anti-mouse Ly-6A/E (Sca-1) Antibody	Biolegend	Cat# 108108; RRID: AB_313345
APC anti-mouse CD117 (c-Kit) Antibody	Biolegend	Cat# 105812; RRID: AB_313221
Pacific Blue™ anti-mouse CD48 Antibody	Biolegend	Cat# 103418; RRID: AB_756140
Brilliant Violet 711™ anti-mouse CD150 (SLAM) Antibody	Biolegend	Cat# 115941; RRID: AB_2629660
CD34 Monoclonal Antibody (RAM34), FITC	eBioscience	Cat# 11-0341-85; RRID: AB_465021
APC/Cyanine7 anti-mouse CD16/32 Antibody	Biolegend	Cat# 101328; RRID: AB_2104158
Streptavidin eFluor™ 450	eBioscience	Cat# 48-4317-82; RRID: AB_10359737
APC/Cyanine7 anti-mouse CD45.1 Antibody	Biolegend	Cat# 110716; RRID: AB_313505
PE/Cy7 anti-mouse CD45.2 Antibody	Biolegend	Cat# 109830; RRID: AB_1186098
FITC anti-mouse CD3 Antibody	Biolegend	Cat# 100204; RRID: AB_312661
PE anti-mouse/human CD45R/B220	Biolegend	Cat# 103208; RRID: AB_312993
Pacific Blue™ anti-mouse/human CD11b	Biolegend	Cat# 101224; RRID: AB_755986
PE/Cy7 anti-mouse CD45.1 Antibody	Biolegend	Car# 110730; RRID: AB_1134168
FITC anti-mouse CD45.2 Antibody	Biolegend	Cat# 109806; RRID: AB_313443
Alexa Fluor® 700 anti-mouse CD45.1 Antibody	Biolegend	Cat# 110724, RRID: AB_493733
Brilliant Violet 785™ anti-mouse CD45.2 Antibody	Biolegend	Cat# 109839, RRID: AB_2562604
PE/Cyanine7 anti-mouse CD150 (SLAM) Antibody	Biolegend	Cat# 115914, RRID: AB_439797
ALKBH5 Recombinant Rabbit Monoclonal Antibody	Thermo Fisher Scientific	Cat# 703570; RRID: AB_2762417
GAPDH (14C10) Rabbit mAb	Cell Signaling Technology	Cat# 2118; RRID: AB_561053
Anti-OGDH antibody	Abcam	Cat# ab137773; RRID: N/A
Anti-rabbit IgG, HRP-linked Antibody	Cell Signaling Technology	Cat# 7074S; RRID: AB_2099233
m6A antibody	Synaptic Systems	Cat# 202003; RRID: AB_2279214
Recombinant Anti-*c*-Myc antibody	Abcam	Cat# ab32072; RRID: AB_731658
Chemicals, peptides, and recombinant proteins		
ACK Lysing Buffer	Thermo Fisher Scientific	Cat# A1049201
Lipofectamine 2000 Transfection Reagent	Thermo Fisher Scientific	Cat# 11668019
Mouse Thrombopoietin (mTPO)	Gemini Bio-product	Cat# 300-351P
Mouse Stem Cell Factor (mSCF)	Gemini Bio-product	Cat# 300-348P
Mouse Flt-3 Ligand (mFlt3L)	Gemini Bio-product	Cat# 300-306P
Mouse Interleukin-3 (mIL3)	Gemini Bio-product	Cat# 300-324P
Benchmark Fetal Bovine Serum (FBS)	Gemini Bio-product	Cat# 100-106
DMEM, high glucose	Thermo Fisher Scientific	Cat# 11965092
RPMI	Thermo Fisher Scientific	Cat# 11875093
X-VIVO™ 15 Serum-free Hematopoietic Cell Medium	Lonza	Cat# BE02-060Q
10% Bovine Serum Albumin in Iscove’s MDM	STEMCELL Technologies	Cat# 9300
MethoCult GF M3434	STEMCELL Technologies	Cat# 03434
iScript cDNA Synthesis Kit	BIO-RAD	Cat# 1708890
iQ SYBR Green Supermix	BIO-RAD	Cat# 1708880
cOmplete, Mini, EDTA-free Protease Inhibitor Cocktail	MilliporeSigma	Cat# 11836170001
Corning™ Matrigel™ GFR Membrane Matrix	Corning	Cat# 354230
SuperSignal™ West Femto Maximum Sensitivity Substrate	Thermo Fisher Scientific	Cat# 34096
8–16% Mini-PROTEAN® TGX Stain-Free™ Protein Gels	BIO-RAD	Cat# 4568104
4-Thiouridine, s4U	Alfa Aesar	Cat# AAJ60679MD
TRIzol™ Reagent	Thermo Fisher Scientific	Cat# 15596018
TURBO™ DNase	Thermo Fisher Scientific	Cat# AM2239
RNAClean XP beads	Beckman Coulter	Cat# A63987
(2S)-Octyl-α-hydroxyglutarate	Cayman	Cat# 16367
Pierce™ Protein A/G Magnetic Beads	Thermo Fisher Scientific	Cat# 88802
RNA Fragmentation Reagents	Thermo Fisher Scientific	Cat# AM8740
DAPI	Thermo Fisher Scientific	Cat# 62248
5-Fluorouracil	InvivoGen	Cat# sud-5fu

**Critical commercial assays**		

EpiQuik m6A RNA Methylation Quantification Kit (Colorimetric)	Epigentek	Cat# P-9005-96
Magnetic mRNA Isolation Kit	New England Biolabs	Cat# S1550S
CellTrace CFSE Cell Proliferation Kit, for flow	Thermo Fisher Scientific	Cat# C34554
RNeasy Mini Kit	QIAGEN	Cat# 74106
MitoProbe™ JC-1 Assay Kit	Thermo Fisher Scientific	Cat# M34152
Seahorse XFe96 FluxPak mini	Agilent	Cat# 102601-100
Seahorse XF Real-Time ATP Rate Assay Starter Pack	Agilent	Cat# 103677-100
Seahorse XF Cell Mito Stress Test Kit	Agilent	Cat# 103015-100
Seahorse XF Glycolysis Stress Test Kit	Agilent	Cat# 103020-100
Mouse Hematopoietic Progenitor (Stem) Cell Enrichment Set	BD Biosciences	Cat# 558451
FITC Annexin V Apoptosis Detection Kit with 7-AAD	BioLegend	Cat# 640922
APC Annexin V Apoptosis Detection Kit with 7-AAD	BioLegend	Cat# 640930
FITC BrdU Flow Kit	BD Biosciences	Cat# 559619
NAD/NADH-Glo™ Assays	Promega	Cat# G9071
Q5® Site-Directed Mutagenesis Kit	New England Biolabs	Cat# E0552S
Succinate Colorimetric Assay Kit	MilliporeSigma	Cat# MAK184
Fumarate Assay Kit	MilliporeSigma	Cat# MAK060
Malate Assay Kit	MilliporeSigma	Cat# MAK067
Mouse B Cell Nucleofector Kit	Lonza	Cat# VPA-1010
MitoTracker™ Green FM Dye	ThermoFisher	Cat# M46750

**Deposited data**		

TimeLapse-seq of murine lineage-depleted BM cells	This paper	GEO: GSE194148

**Experimental models: Cell lines**		

Human: HEK293GP cells	Clontech	Cat# 631458
Experimental models: Organisms/strains		
Mouse: B6.SJL-*Ptprc*^a^*Pepc*^b^/BoyJ	The Jackson Laboratory	JAX: 002014
Mouse: B6.Cg-*Commd10*^*Tg(Vav1-icre)A2Kio*^/J	The Jackson Laboratory	JAX: 008610
Mouse: *Alkbh5*^fl/fl^	Zhou et al., 2021	N/A
Mouse: B6.Cg-Tg(tetO-cre)1Jaw/J	The Jackson Laboratory	JAX: 006234
Mouse: B6.Cg-*Gt(ROSA)26Sor*^*tm1(rtTA∗M2)Jae*^/J	The Jackson Laboratory	JAX: 006965

**Oligonucleotides**		

ON-TARGETplus siRNA-Ythdf2	Horizon	L-058271-01-0005
ON-TARGETplus siRNA-Ogdh	Horizon	L-044219-01-0005
Alkbh5-Forward	CGCGGTCATCAACGACTACC	N/A
Alkbh5-Reverse	ATGGGCTTGAACTGGAACTTG	N/A
Ogdh-Forward	AGGGCATATCAGATACGAGGG	N/A
Ogdh-Reverse	CTGTGGATGAGATAATGTCAGCG	N/A
meRIP-Gapdh-F	AGGTCGGTGTGAACGGATTTG	N/A
meRIP-Gapdh-R	TGTAGACCATGTAGTTGAGGTCA	N/A
meRIP-Ogdh-F	AGGGCATATCAGATACGAGGG	N/A
meRIP-Ogdh-R	CTGTGGATGAGATAATGTCAGCG	N/A
meRIP-Myc-F	GCTTCGAAACTCTGGTGCAT	N/A
meRIP-Myc-R	AATTCCAGCGCATCAGTTCT	N/A
Aco1-Forward	AGAACCCATTTGCACACCTTG	N/A
Aco1-Reverse	AGCGTCCGTATCTTGAGTCCT	N/A
Idh2-Forward	GGAGAAGCCGGTAGTGGAGAT	N/A
Idh2-Reverse	GGTCTGGTCACGGTTTGGAA	N/A
Suclg1-Forward	TGGGCTTGCCCGTCTTTAATA	N/A
Suclg1-Reverse	CTCCGCGTCGATTGCTTCA	N/A
Sdhc-Forward	GCTGCGTTCTTGCTGAGACA	N/A
Sdhc-Reverse	ATCTCCTCCTTAGCTGTGGTT	N/A
Fh1-Forward	GAATGGCAAGCCAAAATTCCTT	N/A
Fh1-Reverse	CGTTCTGTAGCACCTCCAATCTT	N/A
Mdh2-Forward	TTGGGCAACCCCTTTCACTC	N/A
Mdh2-Reverse	GCCTTTCACATTTGCTCTGGTC	N/A
Cs-Forward	GGACAATTTTCCAACCAATCTGC	N/A
Cs-Reverse	TCGGTTCATTCCCTCTGCATA	N/A
Actin-Forward	CTGGCTGGCCGGGACCTGACA	N/A
Actin-Reverse	ACCGCTCGTTGCCAATAGTGATGA	N/A

**Recombinant DNA**		

pMSCV-IRES-GFP	Addgene	Plasmid #20672
pMSCV-*Alkbh5*-IRES-GFP	This paper	N/A
pMSCV-*Alkbh5*-H205A-IRES-GFP	This paper	N/A
pMSCV-*Ogdh*-IRES-GFP	This paper	N/A
pMSCV-*Mll-AF9*-IRES-GFP	Addgene	Plasmid #71443
pCMV-VSV-G	Addgene	Plasmid #8454

**Software and algorithms**		

Fiji	NIH	https://fiji.sc/
Prism v9	GraphPad	https://www.graphpad.com/scientificsoftware/prism/
FlowJo v10	FlowJo, LLC	https://www.flowjo.com/solutions/flowjo
Seahorse Wave Desktop Software	Agilent	N/A

**Other**		

HemaTrue Veterinary Hematology Analyzer	Heska	N/A
Seahorse XFe96 Analyzer	Agilent	N/A
FACSAria	BD	N/A
Amicon® Ultra-15 Centrifugal Filter Unit	MilliporeSigma	Cat# UFC910024
BD Microtainer® Blood Collection Tubes	BD	Cat# 365974

### Resource availability

#### Lead contact

Further information and requests for resources and reagents should be directed to and will be fulfilled by the Lead Contact, Stephanie Halene (stephanie.halene@yale.edu).

#### Materials availability

Plasmids generated in this study are available from the Lead Contact with a completed Materials Transfer Agreement.

### Experimental model and study participant details

#### Mice

All mice were bred and maintained under specific-pathogen-free conditions at the animal facility of Yale University School of Medicine. Animal experiments were performed under protocols approved by the Institutional Animal Care and Use Committee of Yale University. Both female and male mice were used in experiments. Mice of 12–16 weeks of age were used for analysis unless otherwise specified in the text.

We crossed floxed *Alkbh5* mice (Zhou et al., 2021) with B6.Cg-*Commd10*^*Tg(Vav1-icre)A2Kio*^/J (*Vav*-iCre, JAX # 008610) mice to obtain conditional *Alkbh5*-deficient mice.

We crossed Doxycycline (Dox) inducible Cre expression mice rtTA-Cre (B6.Cg-Tg(tetO-cre)1Jaw/J x B6.Cg-*Gt(ROSA)26Sor*^*tm1(rtTA∗M2)Jae*^/J) with *Alkbh5*^fl/fl^ mice to generate the *Alkbh5*^fl/fl^-rtTA-Cre mouse strain. *Alkbh5* was deleted in hematopoietic cells after transplantation by administration of doxycycline in drinking water at 1 mg/mL.

Syngeneic B6.SJL-*Ptprc*^*a*^ *Pepc*^*b*^/BoyJ (Peb3b, JAX: 002014) transplant recipient mice were 8–12 weeks of age. Male and female mice were represented in balanced proportion when in-house colony stock availability necessitated using mixed sex recipients.

Bone marrows of *Alkbh5*^fl/fl^-rtTA (CTA^fl/fl^) and *Alkbh5*^fl/fl^-rtTA-Cre (CTA5^−/−^) mice were transplanted into lethally irradiated (900 cGy) Pep3b mice. Two months after transplantation, recipients were placed on Doxycycline (1 mg/mL) drinking water for one week to delete *Alkbh5* in the hematopoietic system.

#### Generation and analysis of murine *Mll-AF9* leukemia model

Lineage-depleted bone marrow cells were enriched from the bone marrows of 12-week-old Cre-rtTA-*Alkbh5*^fl/fl^ and WT mice and infected with pMSCV-*Mll-AF9*-IRES-GFP retroviruses twice in the presence of 8 μg/mL polybrene at 500 × g for 30 min at room temperature. Cells were then cultured for 3 days with MA9 medium (X-vivo 15 medium+1% BSA+50 ng/mL mSCF+50 ng/mL mTPO+50 ng/mL mFlt3L+20 ng/mL mIL-3). 300,000 GFP^+^ infected cells were retro-orbitally transplanted into sub-lethally irradiated (7 Gy) Pep3b mice. 8 weeks later, 10,000 GFP+ cells were sorted from the bone marrow of recipients and transplanted into secondary recipients irradiate with 7 Gy. The expansion of GFP+ leukemia cells in peripheral blood was monitored and analyzed weekly.

For deletion of *Alkbh5* in the *Mll-AF9* infected Cre-rtTA-*Alkbh5*^fl/fl^ cells (MA9-*Alkbh5*^fl/fl^), cells were cultured in MA9 medium in the presence of 0.5 μg/mL Doxycycline for 4 days.

### Method details

#### Bone marrow isolation

Bone marrows were flushed from tibias and femurs of control and experimental mice and gently dissociated into single cells in PBS with 5% BSA with BD PrecisionGlide Needle (26G×1/2). Cells were lysed by ACK lysing buffer and kept on ice for further use.

#### Western blot

Samples were boiled to denature proteins and separated in 8–16% Mini-PROTEAN TGX Stain-Free Protein Gels. Lysates were transferred to 0.45 μm PVDF membranes with a standard wet transfer system at 90 V for 70 min. Membranes were blocked with 5% skim milk in TBST for 30 min and incubated with primary antibodies overnight at 4°C. Excess antibody was washed away with TBST (50 mM Tris pH 8.0, 150 mM NaCl, 0.1% Tween 20) 3 times. Membranes were incubated with HRP-linked secondary antibody for 1 h at room temperature. After 3 washes, membranes were developed with SuperSignal West Femto Maximum Sensitivity Substrate. Antibodies were applied in 5% skim milk in TBST.

#### Measurement of m^6^A levels on mRNA

Bone marrows were harvested from WT and *vcAlkbh5*^−/−^ mice, and mRNA was isolated using the Magnetic mRNA Isolation Kit (New England Biolabs) following the supplier provided protocol. 150 ng mRNA of each sample was used for the measurement of m^6^A levels using the EpiQuik m^6^A RNA Methylation Quantification Kit following the supplier provided protocol.

#### Flow cytometry

Bone marrow single cell suspensions were blocked with rat anti-mouse CD16/32 antibody for 5 min (except when stained with CD16/32 antibody), followed by staining with antibodies in FACS buffer (5% BSA, 2 mM EDTA in PBS) in the dark at 4°C for 30 min. Cells were washed once with FACS buffer and analyzed. Flow cytometry analysis was performed on FACSymphony (BD Biosciences) instruments, while sorting was performed on the FACS Aria instrument (BD Biosciences). Flow cytometry data were analyzed with FlowJo software (TreeStar).

#### BrdU administration

For cell cycle analysis, BrdU (BD Biosciences) was administrated by intraperitoneal injection (1.5 mg/mouse) every 8 h in sterile saline. 16 h later, mice were sacrificed and bone marrow was isolated and stained with Miltenyi lineage detection Cocktail-Biotin, APC-Cy7 Streptavidin, Sca1-PE, c-Kit-APC, CD48-Pacific blue, CD150-PE/Cy7, CD45.1-AF700, CD45.2-BV785, and fixed and permeabilized following the instructions of the FITC BrdU Flow Kit (BD Biosciences).

#### 5-FU treatment

Mice at the age around 3 months old were injected with 150 mg/kg 5-Fluorouracil (5-FU) intraperitoneally. Mice were sacrificed and analyzed 9 days post 5-FU injection.

#### RNA extraction and quantitative PCR (qPCR)

RNA was isolated using the RNeasy Mini Kit (QIAGEN) per vendor supplied protocol. Reverse-transcription of 1μg RNA was performed using the iScript cDNA Synthesis Kit per standard protocol. Quantitative PCR was carried out in triplicate with target specific primers using iQ SYBR Green Supermix and quantitated using the CFX96 Real-Time System (BIO-RAD).

#### Competitive transplantation and homing efficiency test

For competitive transplantation, 0.5 million CD45.2^+^ bone marrow (BM) cells along with 0.5 million competitor BM cells from CD45.1^+^ Pep3b mice were injected into lethally irradiated (900 cGy) CD45.1^+^ Pep3b recipient mice via retro-orbital injection.

Transplanted recipient mice were monitored daily for signs of distress after transplantation. Sick mice were euthanized and analyzed for engraftment. All other mice were bled every 4 weeks and sacrificed at designated assay endpoints (typically beyond 16 weeks post-transplant; for CTA5 mouse transplantation experiment, recipients were sacrificed 16 weeks after Dox treatment), and engraftment was confirmed by flow cytometry.

For secondary transplantation, BM cells were isolated from the primary recipients 1 million nucleated cells were transplanted into the lethally irradiated (900 cGy) CD45.1^+^ Pep3b secondary recipients via retro-orbital injection. The engraftment rates in PB and BM were analyzed 16 weeks post-transplant.

To test the homing efficiency of BM cells, donor mice were first treated with 150 mg/kg 5-FU. After 4 days, BM cells were isolated from donor mice and incubated with 5 μM CellTrace CFSE for 20 min. After incubation, BM cells were transplanted into lethally irradiated (900 cGy) Pep3b mice (1 million cells per recipient mouse) via retro-orbital injection. After 16 h, recipient mice were sacrificed and CFSE positive cells in their bone marrow and spleens were detected by flow cytometry. Homing efficiency was calculated by multiplying total bone marrow or spleen cells of recipient with the CFSE positivity rate, and divided by the transplanted donor cell number (1 million cells).

#### TimeLapse-seq

Bone marrow cells isolated from WT and *vcAlkbh5*^−/−^ mice were lineage-depleted following the instructions of the Mouse Hematopoietic Progenitor (Stem) Cell Enrichment Set (BD Biosciences).

4 million cells were cultured in HSPC media (DMEM+10% FBS, 50 ng/mL mSCF, 50 ng/mL mTPO, 50 ng/mL mFlt3L, 10 ng/mL mIL3) supplemented with 0.1 mM 4-Thiouridine (s^4^U, Alfa Aesar, AAJ60679MD). The cells were incubated at 37°C for 2 h, and then total RNA was isolated using 1 mL TRIzol. RNA isolated from TRIzol was precipitated in 50% isopropanol supplemented with 1 mM DTT. Genomic DNA was depleted by treating with TURBO DNase and RNA was purified with one volume of Agencourt RNAclean XP beads (Beckman Coulter, Cat #A63987) according to manufacturer’s instructions. 5 μg of total RNA was subjected to 2,2,2-trifluoroethylamine (TFEA, 600 mM final) and sodium periodate (NaIO4, 10 mM final) for 1 h at 45°C followed by reducing treatment for 30 min at 37°C as previously described *(13)*. For each sample, 10 ng of total RNA was used to construct a sequencing library using the Clontech SMARTer Stranded Total RNA-Seq kit (Pico Input) with ribosomal cDNA depletion. Paired-end 100 bp sequencing was performed on an Illumina NovaSeq.

#### m^6^A-RIP-PCR

20 μg total RNA of bone marrow cells were isolated from WT and *vcAlkbh5*^−/−^ mice and purified by Magnetic mRNA Isolation Kit (NEB) to obtain mRNA. An aliquot of total RNA from each sample was kept as input control. Purified mRNAs were then fragmented by RNA Fragmentation Reagents (ThermoFisher Scientific) for 5 min. Fragmented RNA was washed with 1 mL 75% ethanol and spun at 13,500 × g for 10 min and resuspended in RNase-free H_2_O. 30 μL of protein A/G magnetic beads (ThermoFisher Scientific, 88802) were washed twice by IP buffer (150 mM NaCl, 0.1% IGEPAL CA-630, 10 mM Tris-HCl [pH7.5] in nuclease-free H_2_O), resuspended in 500 μL of IP buffer, and tumbled with 2 μg anti-m^6^A antibody (Synaptic Systems, 202003) at 4°C for 2 h. Following 2 washes in IP buffer, the antibody-bead mixture was resuspended in 500 μL of the IP reaction mixture containing fragmented total RNA, 500 μL IP buffer, and 5 μL of SUPERase⋅In RNase Inhibitor and incubated for 2 h at 4°C with head-over-tail rotation.

The RNA reaction mixture was washed twice in 1 mL IP buffer, twice in 1 mL of low-salt IP buffer (50 mM NaCl, 10 mM Tris-HCl [pH 7.5], 0.1% IGEPAL CA-630 in nuclease-free H_2_O), and twice in 1 mL of high-salt IP buffer (500 mM NaCl, 10 mM Tris-HCl [pH 7.5], 0.1% IGEPAL CA-630 in nuclease-free H_2_O). After extensive washing, the m^6^A-enriched fragmented RNA was eluted from the beads in 200 μL of RLT buffer supplied in RNeasy Mini Kit (QIAGEN) for 2 min at room temperature. A magnetic separation rack was used to pull beads to the side of the tube. Supernatant was collected into a new tube, and 400 μL of 100% ethanol was added to it. The mixture was transferred to an RNeasy MiniElute spin column and centrifuged at 12,000 rpm at 4°C for 1 min. The spin column membrane was washed with 500 μL RPE buffer once, then with 500 μL 80% ethanol once, and centrifuged at full speed for 5 min at 4°C to remove the residual ethanol. The m^6^A-enriched RNA was eluted with 14 μL RNase-free H_2_O.

Reverse-transcription and qPCR analysis were performed following the instruction of the kit of iScript cDNA Synthesis Kit (BIO-RAD) and iQ SYBR Green Supermix (BIO-RAD).

#### NAD/NADH-Glo assay

WT and *vcAlkbh5*^−/−^ mice were sacrificed and their lineage-depleted BM was isolated following the instructions of the Mouse Hematopoietic Progenitor (Stem) Cell Enrichment Set. 20,000 lineage depleted cells of each sample were resuspended in 50 μL PBS to measure NAD+ and NADH separately following the protocol of NAD/NADH-Glo Assays (Promega).

#### Sample preparation for LC-MS/MS analysis

50 μL mouse plasma was collected from each mouse, 100 μL of acetonitrile (ACN) and 5 μL of two internal standards (C5-α-KG and C5-D-2-HG, 10 μM) was added. The tubes were subsequently centrifuged at 20,000 ×g for 10 min to remove precipitated proteins. Then, the supernatant was transferred to another clean tube and dried in a vacuum concentrator at room temperature.

#### Chiral derivatization

To separate the two enantiomers of 2-HG without a chiral stationary phase, *N*-(*p*-toluenesulfonyl)-L-phenylalanyl chloride (TSPC) was used to derivatize the enantiomers followed with LC-MS/MS analysis. The derivatization procedure was followed as previously published (Cheng et al., 2015). Briefly, 100 μL TSPC (2.5 mM in ACN) and 2 μL pyridine were added to the dry residue. The mixture was then incubated at 40°C for 0.5 h. After the derivatization reaction, the mixture was dried in a vacuum concentrator at room temperature and then reconstituted in 50 μL 50% aqueous ACN. Subsequently, 5 μL of each sample was subjected to LC-MS/MS analysis.

#### LC-MS/MS analysis

The quantification of TSPC labeled D/L-2HG and α-KG were performed on the LC-ESI-MS/MS system consisting of an Agilent 6490 triple quad LC-MS (Agilent technologies, USA) with an electrospray ionization source and an Agilent 1290 Infinity HPLC system (Agilent technologies, USA). The HPLC separation was performed on an Agilent ZORBAX SB-C18 column (150 mm × 4.6 mm i.d., 5μm, Agilent technologies, USA) at 40°C. Ammonium formate aqueous solution (2 mM, solvent A) and pure ACN (solvent B) were employed as the mobile phase. A gradient of 3 min 5% B, 5 min 5–20% B, 10 min 20% B, 11 min 20–90% B, 13 min 90% B, 14 min 90-5% B, and 20 min 5% B was used. The flow rate of mobile phase was set at 1.0 mL/min.

The mass spectrometry detection was performed using multiple reaction monitoring (MRM) under negative ion mode. The mass transitions (precursor ions→ product ions) were 448.1→ 318.1 and 448.1→ 155.1 for TSPC labeled 2HG, 453.1→ 318.1 and 453.1→ 155.1 for TSPC labeled C5-2HG, 145.1→ 101.1 and 145.1→ 57.1 for α-KG, and 150.1→ 105.1 and 150.1→ 60.1 for C5-α-KG. The MRM parameters of all analytes were optimized to achieve maximal detection sensitivity.

#### Metabolic assays

For the Real-Time ATP Rate Assay, lineage-depleted BM cells of WT and *vcAlkbh5*^−/−^ mice were plated on Matrigel-coated Seahorse Bioanalyzer XFe96 culture plates (200,000 cells/well) in assay media (Seahorse XF DMEM Medium, pH 7.4 supplemented with 10 mM glucose, 1 mM pyruvate, 2 mM glutamine). Oligomycin (1.5 μM), and rotenone/antimycin A (0.5 μM) were injected to measure the production rates of ATP, with three measurements performed after each injection. Oligomycin (ATP synthase blocker) was used to measure ATP turnover and to determine proton leak; Rotenone (inhibitor of complex I) and Antimycin A (a blocker of complex III) were injected to completely shut down mitochondrial respiration, to confirm that any changes observed in respiration were mitochondrial. Data were analyzed by WAVE software (Agilent).

For the Cell Mito Stress Test, lineage-depleted bone marrow cells of WT and *vcAlkbh5*^−/−^ mice or Mll-AF9 leukemia cells were plated on Matrigel-coated Seahorse Bioanalyzer XFe96 culture plates (150,000 cells/well or 100,000 cells/well) in assay media (Seahorse XF DMEM Medium, pH 7.4 supplemented with 10 mM glucose, 1 mM pyruvate, 2 mM glutamine). Oligomycin (1.5 μM), FCCP (1 μM), and rotenone/antimycin A (0.5 μM) were injected in order. OCR and ECAR were measured to determine the mitochondrial function, with three measurements performed after each injection. Oligomycin (ATP synthase blocker) was used to measure ATP turnover and to determine proton leak; the mitochondrial uncoupler carbonyl cyanide 4-[trifluoromethoxy] phenylhydrazone (FCCP) was used to measure maximum respiratory function (maximal OCR). Rotenone (inhibitor of complex I) and Antimycin A (a blocker of complex III) were injected to completely shut the mitochondrial respiration down, to confirm that any changes observed in respiration were mitochondrial. Data were analyzed by WAVE software.

#### Measurement of metabolites in murine plasma

Murine blood samples were collected by retro-orbital bleeding into BD Microtainer Blood Collection Tubes, and centrifuged for 10 min at 12,000 × g at 4°C. Transparent plasmas were collected and stored at −80°C until use for the experiment.

20 μL plasma of each sample was used to measure different metabolites (Succinate, Fumarate, Malate) following the instructions in respective kits (MilliporeSigma).

#### Apoptosis assay and mitochondrial health test

For apoptosis assay of competitive transplanted mice, BM cells from WT and *vcAlkbh5*^−/−^ groups were stained with Miltenyi lineage detection Cocktail-Biotin, APC-Cy7 Streptavidin, Sca1-PE, c-Kit-APC, CD48-Pacific blue, CD150-PE/Cy7, CD45.1-AF700, CD45.2-BV785 first, and then washed with FACS buffer, followed by FITC Annexin V Apoptosis Detection Kit and 7-AAD, and then analyzed by flow cytometry per standard protocol.

For apoptosis assay of *Mll-AF9* leukemia cells, cells were stained with APC Annexin V Apoptosis Detection Kit and 7-AAD, and then analyzed by flow cytometry per standard protocol.

For mitochondrial health test, the control tubes of WT and *vcAlkbh5*^−/−^ BM cells were treated with 50 μM CCCP for 5 min at 37°C. After that, control tubes and experimental tubes were both incubated with 1 μM JC-1 for 15 min at 37°C following the steps provided by MitoProbe JC-1 Assay Kit. Finally, cells were washed and analyzed by flow cytometry.

#### MitoTracker green staining

Isolate bone marrow from WT and *vcAlkbh5*^−/−^ mice, incubate cells with desired antibody for 30 min. Wash cells with FACS buffer and then incubate cells with 500 μL PBS and 1 μL MitoTracker Green dye for 30 min at 37°C, protected from light. Add 7-AAD to the buffer and analyze cells by flow cytometry.

#### Electron microscope

Bone marrow cells from WT and *vcAlkbh5*^−/−^ mice were lineage-depleted following the instructions of Mouse Hematopoietic Progenitor (Stem) Cell Enrichment Set (BD Biosciences). The lineage-depleted BM cells were fixed in 2.5% glutaraldehyde in 0.1M cacodylate buffer (pH 7.4), then post-fixed in 1% OsO4 in the same buffer at room temperature for 1 h. After staining *en bloc* with 2% aqueous uranyl acetate for 30 min, cells were dehydrated in a graded series of ethanol to 100% and finally embedded in EMbed 812 resin. Blocks were then polymerized in 60°C oven for 24 h. Thin sections (60 nm) were cut by a Leica ultramicrotome (UC7) and post-stained with 2% uranyl acetate and lead citrate. Sample grids were examined with a FEI Tecnai G2 transmission electron microscope at 80 kV of the accelerating voltage, digital images were recorded with an Olympus Morada CCD camera and iTEM imaging software.

#### L-2-HG treatment and seahorse assay

MOLM13 cells (a gift from Michael G. Kharas’ lab at Memorial Sloan Kettering Cancer Center) were cultured with RPMI with 10% FBS and treated with different concentrations of L-2-HG ((2S)-Octyl-α-hydroxyglutarate) for 2 days. Then cells were replated into Matrigel-coated Seahorse Bioanalyzer XFe96 culture plates at the concentration of 100,000 cells/well in assay media (Seahorse XF DMEM Medium, pH 7.4 supplemented with 10 mM glucose, 1 mM pyruvate, 2 mM glutamine). Oligomycin (1.5 μM), and rotenone/antimycin A (0.5 μM) were injected to measure the production rates of ATP, three measurements were performed after each injection. Data were analyzed by WAVE software (Agilent).

Murine lineage-depleted BM cells were isolated from WT murine BM and enriched by Mouse Hematopoietic Progenitor (Stem) Cell Enrichment. The lineage-depleted BM cells were cultured with HSPC culture medium (DMEM+10% FBS, 50 ng/mL mSCF, 50 ng/mL mTPO, 50 ng/mL mFlt3L, 10 ng/mL mIL3) for 2 days. The suspension cells were replated into Matrigel-coated Seahorse Bioanalyzer XFe96 culture plates at the concentration of 150,000 cells/well in assay media (Seahorse XF DMEM Medium, pH 7.4 supplemented with 10 mM glucose, 1 mM pyruvate, 2 mM glutamine). Oligomycin (1.5 μM), and rotenone/antimycin A (0.5 μM) were injected to measure the production rates of ATP, three measurements were performed after each injection. Data were analyzed by WAVE software.

#### Plasmids

Murine *Alkbh5* cDNA was amplified from the testis of wildtype mouse and cloned into pMSCV-IRES-GFP vector (pMIG-*Alkbh5*) using EcoRI and XhoI restriction sites, and catalytically dead *Alkbh5*-H205A (pMIG-*Alkbh5*-H205A) was generated from pMIG-*Alkbh5* plasmid following the protocol provided by Q5 Site-Directed Mutagenesis Kit (New England Biolabs). Murine *Ogdh* cDNA was amplified from the kidney of a wildtype mouse and cloned into pMSCV-IRES-GFP vector (pMIG-*Ogdh*) using EcoRI and XhoI restriction sites. The corresponding primers used are as follows:

wildtype Alkbh5 (forward, 5ʹ-ttatgaattcATGGCGGCCGCCAGCGGCTAC-3ʹ; reverse, 5ʹ-actctcgagTCAGTGTCTCCTCATCTTCACCTTGCGGGTGG-3ʹ); Alkbh5-H205A (forward, 5ʹ-CATCGTGTCCGCCGTTGACCCCATCCACATCTTCG-3ʹ; reverse, 5ʹ-CAGCCGCCGGGCTGGTAG-3ʹ);

*Ogdh* (forward, 5ʹ-gccggaattcATGTTTCATTTAAGGACTTGTGCTGCTAAG-3ʹ; reverse, 5ʹ-ccgctcgagCTAAGAGAATTTCTTGAATGCGTCCAGG-3ʹ).

Uppercase letters are nucleotides matched with the targeted gene sequence, red uppercase letters introduced the mutated nucleotides, while lowercase letters are the restriction endonuclease recognition sites and overhang nucleotides for cloning purposes. All constructs were confirmed by Sanger sequencing.

#### Virus transduction and rescue experiments

Retroviral supernatants were generated via co-transfection of 293GP cells (Clontech, Cat # 631458) with pCMV-VSVG and pMIG-empty or pMIG-*Alkbh5* or pMIG-*Alkbh5*-H205A or pMIG-*Ogdh*, or pMIG-*Mll-AF9* plasmids, followed by spin-concentration using Amicon Ultra-15 columns per supplier instructions (MilliporeSigma).

WT and *vcAlkbh5*^−/−^ mice were pre-treated with 5-Fluorouracil at 150 mg/kg 4 days prior to bone marrow isolation. Isolated bone marrow cells were cultured overnight and then infected with viral supernatants at equivalent multiplicity of infection (MOI) via spinoculation (1000 ×g for 45 min at 25°C) with addition of 8 μg/mL polybrene (Sigma). A second transduction was performed on the following day. After transduction, cells were cultured in HSPC culture medium (DMEM+10% FBS, 50 ng/mL mSCF, 50 ng/mL mTPO, 50 ng/mL mFlt3L, 20 ng/mL mIL3) for one day.

For rescue experiment in competitive transplantation assay, unsorted 0.5 million virally transduced bone marrow cells were co-transplanted with 0.5 million competitor (CD45.1+) bone marrow cells into lethally irradiated Pep3b recipients (900 cGy). Engraftment was measured 16-week post transplantation.

For rescue experiment in homing efficiency test, lineage-depleted BM cells were infected with the virus to overexpress *Alkbh5*, *Ogdh*, and empty vector by spinoculation twice on day 0 and day 1. The infected cells were cultured in HSPC media and sorted for GFP positive cells on day 2. The sorted cells were then transplanted into lethally irradiated Pep3b recipients (900 cGy). Engraftment was measured 16-h post transplantation.

#### Knockdown experiment with siRNA transfection

For *Ythdf2* knockdown in lineage-depleted bone marrow cells, electroporation was performed with Amaxa Mouse B Cell Nucleofector Kit. Transfected cells were cultured for 2 days with HSPC media. RNA was extracted and gene expression level of *Ogdh* was measured by qPCR.

For *Ogdh* knockdown in MA9 cells, electroporation was performed with Amaxa Mouse B Cell Nucleofector Kit. Transfected cells were plated in MethoCult M3434 at the concentration of 1,000 cells/35 mm dish. Colonies were evaluated and scored after incubation at 37°C and 5% CO_2_ for 7 days.

Colony-forming unit (CFU) assay of MA9 cells.

For CFU assay, sorted GFP+ MA9-WT and MA9-*Alkbh5*^−/−^ cells were plated in MethoCult M3434 at the concentration of 1,000 cells/35 mm dish. Colonies were evaluated and scored after incubation at 37°C and 5% CO_2_ for 7 days.

L-2-HG effect on cell viability measured by CellTiter-Glo assay and Annexin V staining.

25,000 MOLM13 cells were seeded into 96-well plate and cultured for 2 days, with the concentration of L-2-HG 3.125 μM, 6.25 μM, 12.5 μM, 25 μM, 50 μM, 100 μM, 200 μM in 100 μL RPMI medium with 10% FBS.

For CellTiter-Glo assay, after 2 days of culture, the medium containing cells was transferred to a new 96-well white plate and added with 100 μL CellTiter Glo 2.0 solution to each well. The contents were mixed for 2 min to induce cell lysis and read by the Bio-Tek plate reader to record luminescence.

For Annexin V staining, after 2 days of culture, the cells were stained with FITC Annexin V Apoptosis Detection Kit and 7-AAD, and then analyzed by flow cytometry per standard protocol.

#### Data analysis

##### TimeLapse-seq mutation calling

Filtering and alignment to the mouse mm10 (GRCm38) genome were performed essentially as previously described.^36^ Briefly, reads were trimmed of adaptor sequences with Cutadapt v1.16 and aligned to the mm10 genome with HISAT2 with default parameters and --mp 4,2. Reads aligning to annotated transcripts were quantified with HTSeq (https://pypi.org/project/HTSeq/) htseq-count. SAMtools v1.5 was used to collect only uniquely mapped read pairs (SAM flag = 83/163 or 99/147). For mutation calling, T-to-C mutations were not considered if the base quality score was less than 40 and the mutation was within 3 nucleotides from the read’s end. Sites of likely single-nucleotide polymorphisms (SNPs) and alignment artifacts (identified with bcftools) and sites of high mutation levels in the non-s4U treated controls (binomial likelihood of observation p < 0.05) were not considered in mutation calling. Normalization scale factors were calculated with edgeR using calcNormFactors (method = ‘upperquartile’). Browser tracks were made using STAR (version 2.5.3a) and visualized in IGV (https://software.broadinstitute.org/software/igv/).

##### TimeLapse-seq estimation of kinetic parameters

All samples treated with 2-h s^4^U feeds were modeled with the same Poisson model, similar to what was previously described.^36^ The -s^4^U samples were used as unlabeled controls to identify SNPs and determine the background mutation rate attributed reverse transcription mistakes, sequencing error, or other sources. The number of T-to-C mutations observed (*tc*) was modeled as a mixture of two Poisson distributions of either true TimeLapse or background mutations parametrized on the log scale and depends on the fraction of new RNA for the transcript (*θ*). The probability mass function of the model is:

$$f\left(tc|\lambda_{n},\lambda_{o}\right)=\theta PoissonLog\left(tc|\lambda_{n}\right)+\left(1-\theta \right)PoissonLog\left(tc|\lambda_{o}\right)$$

where λ_*n*_ is the TimeLapse mutation rate in new transcripts and λ_*o*_ is the background mutation rate.

To estimate these parameters, we used a Bayesian hierarchical modeling approach using RStan software (Version 2.19.3) that implements no-U-turn Markov Chain Monte Carlo (MCMC) sampling. We designed non-centered hierarchical models to estimate global TimeLapse mutation rate ($\bar{\lambda }$_*n*[*j*]_) for the *j*^th^ treatment condition while also allowing for variability by estimating gene specific mutation probabilities (λ_*n*[*j,s*]_). For the background mutation rate, we estimated a single global parameter ($\bar{\lambda }$_*o*_) while allowing for local variation among genes by estimating gene-specific mutation probabilities (λ_*o*[*s*]_). We used weakly informative priors for global mutation rates on the logistic scale which covered the range of previously observed mutation rates. The gene-specific mutation rates were found by estimating a standard deviation (*σ*) for each global parameter and a gene-specific *Z* score (*z*). Finally, s^4^U-treated and -untreated samples are indicated by *I* where *I* = 1 if sample *c* is labeled with s^4^U and 0 if the sample is unlabeled.

Global parameter priors:

$${\bar{\lambda }}_{o}∼\text{Normal}\left(-3,1.5\right)$$

$${\bar{\lambda }}_{n\left[j\right]}∼\text{Normal}\left(-1,1.5\right)$$

$$\sigma_{o}∼\text{HalfCauchy}\left(0,1.5\right)$$

$$\sigma_{n\left[j\right]}∼\text{HalfCauchy}\left(0,1.5\right)$$

$$I_{\left[c\right]}=\left\{ \begin{array}{c} 0,\text{if}\;c\in \text{controls}\\ 1,\text{otherwise} \end{array}\right.$$

$$s\in \left\{1,2,\ldots ,n_{\text{gene}}\right\}$$

$$j\in \left\{1,2,\ldots ,n_{conditions}\right\}$$

Localparameterpriors:

$$z_{o\left[s\right]}\;∼\;\text{Normal}\left(0,1.5\right)$$

$$z_{n\left[j,s\right]}\;∼\;\text{Normal}\left(0,1.5\right)$$

$$\lambda_{o\left[s\right]}={\bar{\lambda }}_{o}+\sigma_{o}z_{o\left[s\right]}$$

$$\lambda_{n\left[j,s\right]}={\bar{\lambda }}_{n\left[j\right]}+\sigma_{n\left[j\right]}z_{n\left[j,s\right]}$$

For reads $i\in \left\{1,2,\ldots ,n_{\left[s\right]}\right\}$:

$$f\left({tc}_{\left[i\right]}|\theta_{\left[j,s\right]},\lambda_{n\left[j,s\right]},\lambda_{o\left[s\right]}\right)=$$

$$\prod_{i=1}^{n_{\left[s\right]}}\left(I_{\left[c\right]}\theta_{\left[j,s\right]}PoissonLog\left(y_{\left[i\right]}|\lambda_{n\left[j,s\right]}\right)+\left(1-I_{\left[c\right]}\theta_{\left[j,s\right]}\right)PoissonLog\left(y_{\left[i\right]}|\lambda_{o\left[s\right]}\right)\right)$$

Within the same model, fraction new estimates are then converted into degradation rates. We assume an exponential model relating the new fraction of transcripts at the *s*^th^ gene and the observed turnover rate constant for RNA (*k*_*deg*[*s*]_) such that

$$\theta_{\left[s\right]}=1-e^{\left(-{k_{\deg}}_{\left[s\right]}t\right)}$$

where *t* is the s^4^U labeling time of the experiment. As steady state RNA depends on the ratio of the synthesis rate (*k*_*syn*_) to the degradation rate (*k*_*deg*_), we define *k*_*syn*_ as

$$k_{syn\left[j,s\right]}=N_{\left[j,s\right]}k_{\deg\left[j,s\right]}$$

where *N*_[*j*,*s*]_ is the normalized read count in condition *j* aligned to gene *s*. Because the concentration of total RNA is equal to the ratio of *k*_*syn*_ to *k*_*deg*_, the change in total RNA depends on the change in synthesis (L2FC *k*_*syn*_) and degradation (L2FC *k*_*deg*_).

$$L2FC\;N={{L2FC\;k}_{syn}-L2FC\;k}_{\deg}$$

Consequently, the difference in magnitude between L2FC *k*_*deg*_ and L2FC *k*_*syn*_ cannot exceed the change in total RNA. The fraction of change in total RNA attributed to degradation at gene *s* (*frac*_*deg*[*s*]_) is defined as

$${frac}_{\deg\left[s\right]}=\left(\frac{\left|{L2FC\;k}_{\deg\left[s\right]}\right|-\left|{L2FC\;k}_{syn\left[s\right]}\right|}{L2FC\;N_{\left[s\right]}}+1\right)/2$$

where the change in expression between WT and *vcAlkbh5*^−/−^ (L2FC *N*_[*s*]_) were determined by DESeq2. This definition of *frac*_*deg*_ restricts its value to a scale between 0 and 1 and is interpretable as a measure for what fraction of change in steady state RNA levels is attributable to changes in stability of the transcript.

This model converged well on the set of genes that had at least one read in all samples. In all cases, we used the median value of the posterior distribution as a point estimate for the true value. We extracted the 80% confidence interval of *frac*_*deg*_ to identify genes whose change could be attributed primarily to changes in stability (*k*_*deg*_) or synthesis (*k*_*syn*_). If the 80% credible interval does not overlap 0.5, we called the gene as confidently driven by changes in stability (*frac*_*deg*_ > 0.5) or synthesis (*frac*_*deg*_ < 0.5).

#### GO enrichment analysis

Destabilized genes determined by TimeLapse-seq were further analyzed for GO enrichment analysis on WebGestalt (http://www.webgestalt.org), using Over-Representation Analysis (ORA) as method to define the biological process and enriched KEGG pathways.

### Quantification and statistical analysis

All statistical analyses were performed using GraphPad Prism 9 (GraphPad Software). Significance was calculated using two-tailed, unpaired Student’s t-test. n.s. not significant, ^∗^p < 0.05, ^∗∗^p < 0.01, ^∗∗∗^p < 0.001, ^∗∗∗∗^p < 0.0001. All additional details can be found in the figure legends.

## Data Availability

• TimeLapse-seq data generated in this study have been deposited at GEO and are publicly available as of the date of publication. Accession numbers are listed in the Key resources table. • This paper does not report original code. • Any additional information required to reanalyze the data reported in this paper is available from the lead contact upon request.

## Supplemental information

Document S1. Figures S1–S6

Document S2. Article plus supplemental information
