# Supplementary material for: ALKBH5 modulates hematopoietic stem and progenitor cell energy metabolism through m6A modification-mediated RNA stability control
Source: Cell Rep. 2023 Sep 23;42(10):113163. doi: 10.1016/j.celrep.2023.113163 (PMC10636609; doi:10.1016/j.celrep.2023.113163)
Supplement: Document S1. Figures S1–S6 [file mmc1.pdf]

Cell Reports, Volume *42*

Supplemental information

ALKBH5 modulates hematopoietic stem and progenitor cell energy metabolism through m^{6}A modification-mediated RNA stability control

Yimeng Gao, Joshua T. Zimmer, Radovan Vasic, Chengyang Liu, Rana Gbyli, Shu-Jian Zheng, Amisha Patel, Wei Liu, Zhihong Qi, Yaping Li, Raman Nelakanti, Yuanbin Song, Giulia Biancon, Andrew Z. Xiao, Sarah Slavoff, Richard G. Kibbey, Richard A. Flavell, Matthew D. Simon, Toma Tebaldi, Hua-Bing Li, and Stephanie Halene

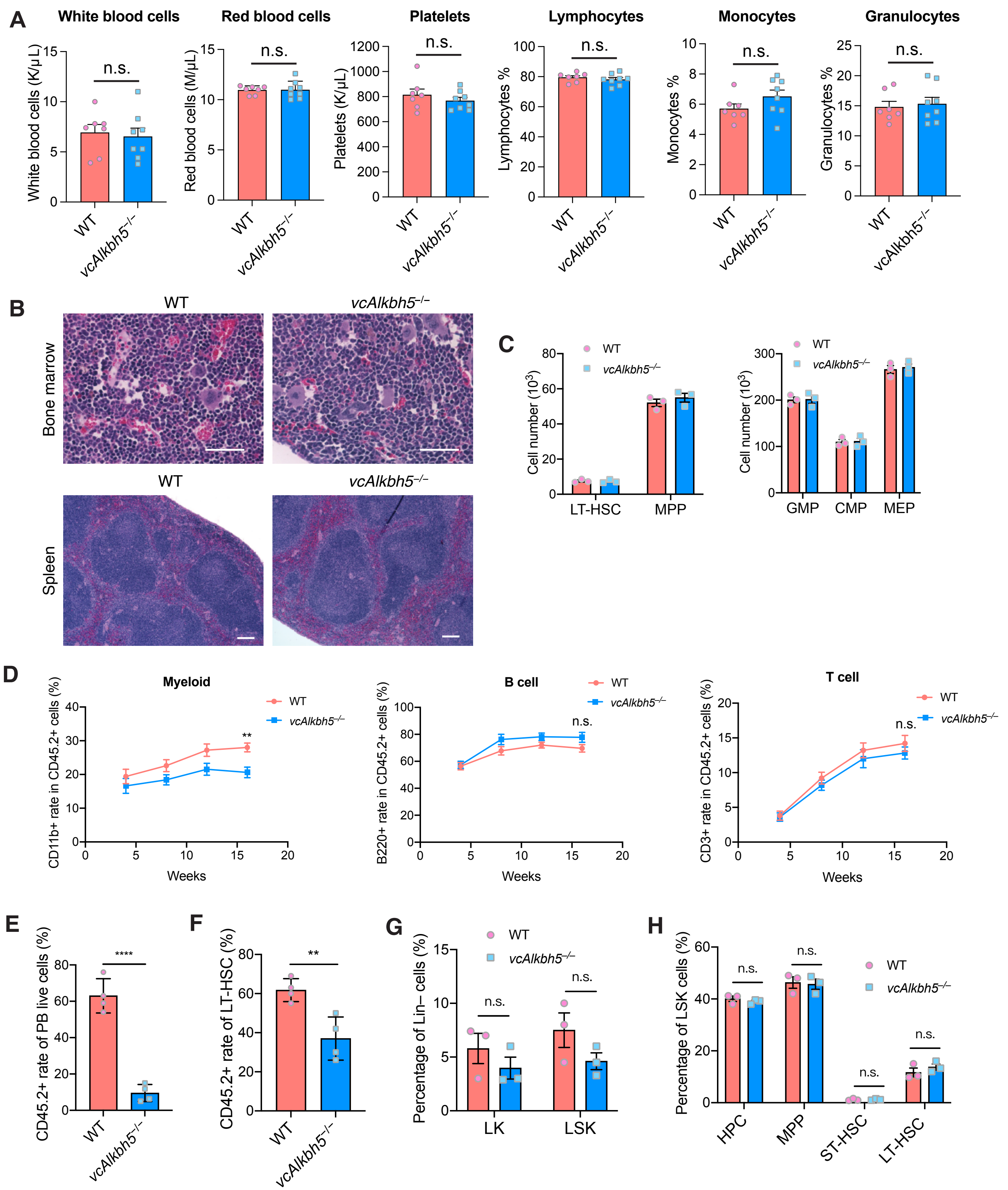
A
White blood cells
Red blood cells
Platelets
Lymphocytes
Monocytes
Granulocytes
White blood cells (K/μL)
Red blood cells (M/μL)
Platelets (K/μL)
Lymphocytes %
Monocytes %
Granulocytes %
n.s.
WT
vcAlkbh5–/–
B
WT
vcAlkbh5–/–
Bone marrow
Spleen
C
Cell number (10³)
LT-HSC
MPP
GMP
CMP
MEP
D
Myeloid
B cell
T cell
CD11b+ rate in CD45.2+ cells (%)
B220+ rate in CD45.2+ cells (%)
CD3+ rate in CD45.2+ cells (%)
Weeks
**
E
CD45.2+ rate of PB live cells (%)
****
F
CD45.2+ rate of LT-HSC (%)
G
Percentage of Lin– cells (%)
LK
LSK
H
Percentage of LSK cells (%)
HPC
MPP
ST-HSC
LT-HSC

Figure S1. ALKBH5 is dispensable for hematopoiesis at steady state. Related to Figure 1.

(A) Peripheral blood counts of WT and *vcAlkbh5*$^{-/-}$ mice (WT n=7, *vcAlkbh5*$^{-/-}$ n=8). (B) Histology of bone marrow and spleen of WT and *vcAlkbh5*$^{-/-}$ mice stained with H&E. Scale bar, 50 µm for the bone marrow, 100 µm for the spleen. (C) Absolute cell number per mouse of LT-HSC, MPP, GMP, CMP and MEP cells measured by flow cytometry. (D) Relative contribution of CD45.2^{+} WT and *vcAlkbh5*$^{-/-}$ *cells* to each blood lineage at specified time points post competitive transplantation. (E) Secondary transplantation PB. (F) Secondary transplantation BM LT-HSC. (G) LK and LSK cell recovery 9 days after 5-FU treatment. (H) HSC and MPP distribution within LSK population 9 days after 5-FU treatment.

Figure S2

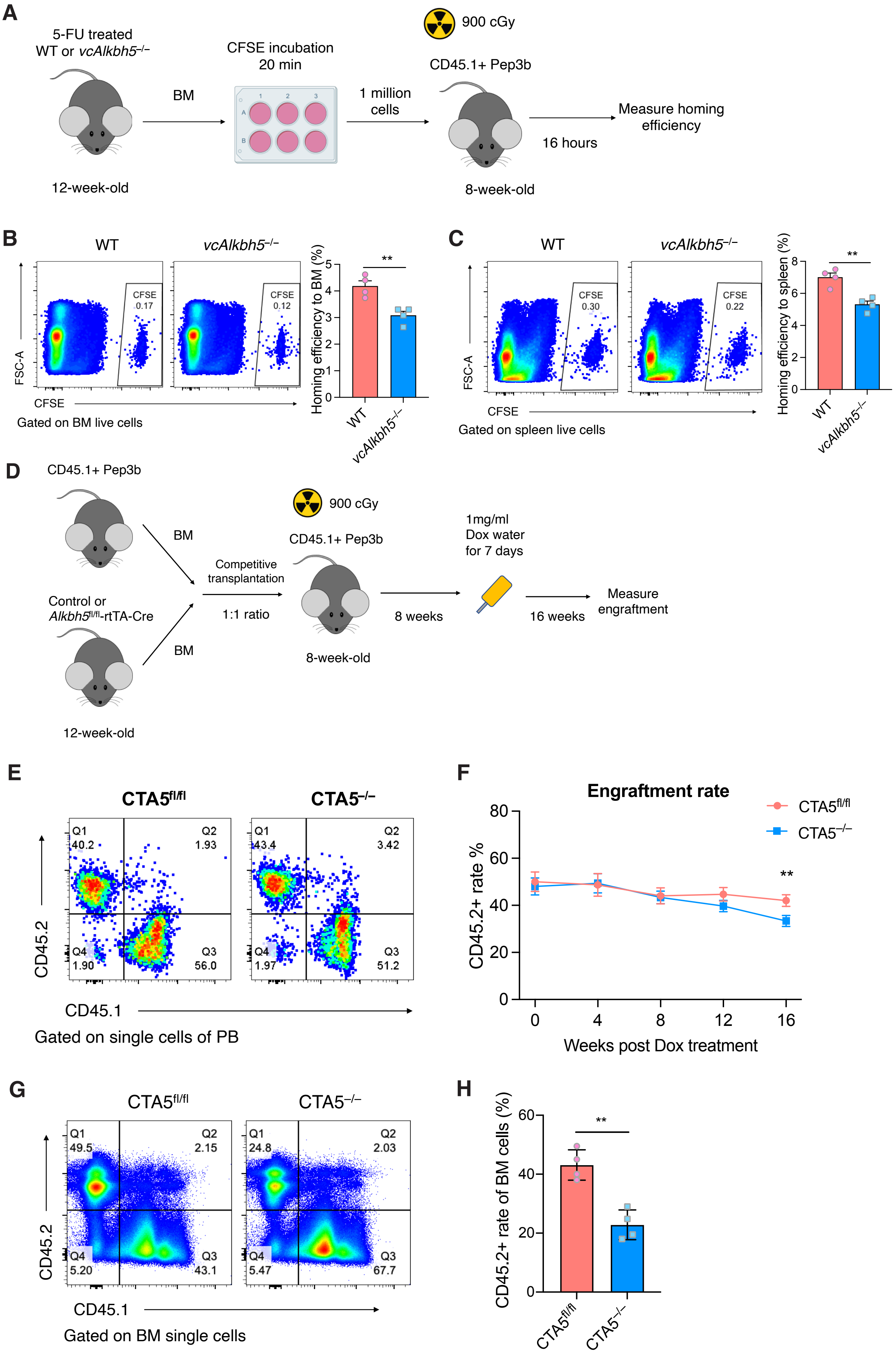
A
5-FU treated
WT or vcAlkbh5–/–
12-week-old
BM
CFSE incubation
20 min
1 million
cells
900 cGy
CD45.1+ Pep3b
8-week-old
16 hours
Measure homing
efficiency
B
WT
vcAlkbh5–/–
CFSE 0.17
CFSE 0.12
FSC-A
CFSE
Gated on BM live cells
Homing efficiency to BM (%)
**
WT
vcAlkbh5–/–
C
WT
vcAlkbh5–/–
CFSE 0.30
CFSE 0.22
FSC-A
CFSE
Gated on spleen live cells
Homing efficiency to spleen (%)
**
WT
vcAlkbh5–/–
D
CD45.1+ Pep3b
BM
Control or
Alkbh5fl/fl-rtTA-Cre
BM
12-week-old
Competitive
transplantation
1:1 ratio
900 cGy
CD45.1+ Pep3b
8-week-old
8 weeks
1mg/ml
Dox water
for 7 days
16 weeks
Measure
engraftment
E
CTA5fl/fl
CTA5–/–
Q1 40.2
Q2 1.93
Q4 1.90
Q3 56.0
Q1 43.4
Q2 3.42
Q4 1.97
Q3 51.2
CD45.2
CD45.1
Gated on single cells of PB
F
Engraftment rate
CTA5fl/fl
CTA5–/–
CD45.2+ rate %
**
Weeks post Dox treatment
G
CTA5fl/fl
CTA5–/–
Q1 49.5
Q2 2.15
Q4 5.20
Q3 43.1
Q1 24.8
Q2 2.03
Q4 5.47
Q3 67.7
CD45.2
CD45.1
Gated on BM single cells
H
CD45.2+ rate of BM cells (%)
**
CTA5fl/fl
CTA5–/–

Figure S2. Loss of ALKBH5 leads to homing defects and cell autonomous competitive disadvantage. Related to Figure 1.

(A) Schematic detailing homing test of WT and *vcAlkbh5*$^{-/-}$ BM cells. (B) Determination of homing efficiency to recipient BM (B) and spleen (C) by flow cytometry; homing efficiency was calculated taking into account transplanted cell number and contribution to total BM or spleen cell number at time of harvest (n=4 of each group). (D) Schematic of competitive transplantation of CTA5$^{fl/fl}$ and CTA5$^{-/-}$ BM cells. (E) Competitive engraftment of CTA5$^{fl/fl}$ versus CTA5$^{-/-}$ cells in the peripheral blood of recipients before Dox treatment. (F) Kinetics of the engraftment rate of CTA5$^{fl/fl}$ and CTA5$^{-/-}$ mice in PB for 16 weeks after Dox treatment (n=4 of each group). (G and H) Engraftment rate of CD45.2^{+} cells in the bone marrow 16 weeks after Dox treatment, as measured by flow cytometry (G) and quantified in (H). Data are represented as mean ± SEM and representative of at least two independent experiments; p values were calculated using two-tailed Student's t test. ** $p<0.01$.

Figure S3

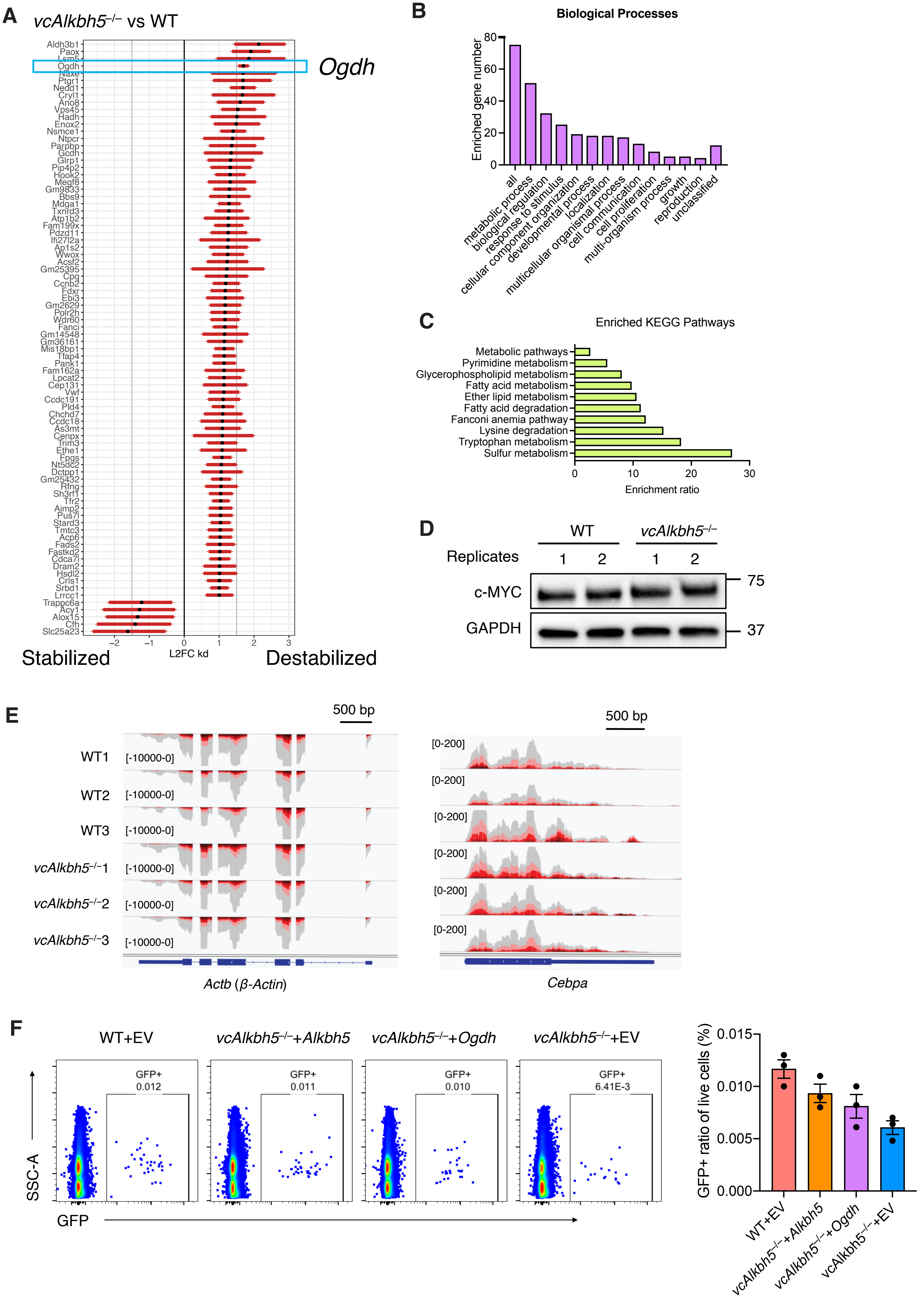
A
vcAlkbh5–/– vs WT
Ogdh
Stabilized
L2FC kd
Destabilized
B
Biological Processes
Enriched gene number
all
metabolic process
biological regulation
response to stimulus
cellular component organization
developmental process
localization
multicellular organismal process
cell communication
cell proliferation
multi-organism process
growth
reproduction
unclassified
C
Enriched KEGG Pathways
Metabolic pathways
Pyrimidine metabolism
Glycerophospholipid metabolism
Fatty acid metabolism
Ether lipid metabolism
Fatty acid degradation
Fanconi anemia pathway
Lysine degradation
Tryptophan metabolism
Sulfur metabolism
Enrichment ratio
D
WT
vcAlkbh5–/–
Replicates
c-MYC
GAPDH
75
37
E
500 bp
WT1
WT2
WT3
vcAlkbh5–/–1
vcAlkbh5–/–2
vcAlkbh5–/–3
[-10000-0]
[0-200]
Actb (β-Actin)
Cebpa
F
WT+EV
vcAlkbh5–/–+Alkbh5
vcAlkbh5–/–+Ogdh
vcAlkbh5–/–+EV
GFP+
0.012
0.011
0.010
6.41E-3
SSC-A
GFP
GFP+ ratio of live cells (%)

Figure S3. Loss of ALKBH5 destabilizes mRNAs of metabolic pathway genes. Related to Figure 3.

(A) SigmaPlot of changes in RNA decay determined by TimeLapse-seq. Black point represents the median. Red bar represents the 80% credible interval. (B) GO analysis summarizing the enriched gene numbers in each biological process of destabilized genes. (C) Enriched KEGG pathways of destabilized genes in *vcAlkbh5*$^{-/-}$ lineage-depleted BM cells. (D) C-Myc rotein levels in WT and *vcAlkbh5*$^{-/-}$ BM measured by immunoblot. (E) TimeLapse-seq tracks depicting the coverage of *Actb* and *Cebpa* reads. (F) Measurement of homing cells in the recipient mice after rescue experiment.

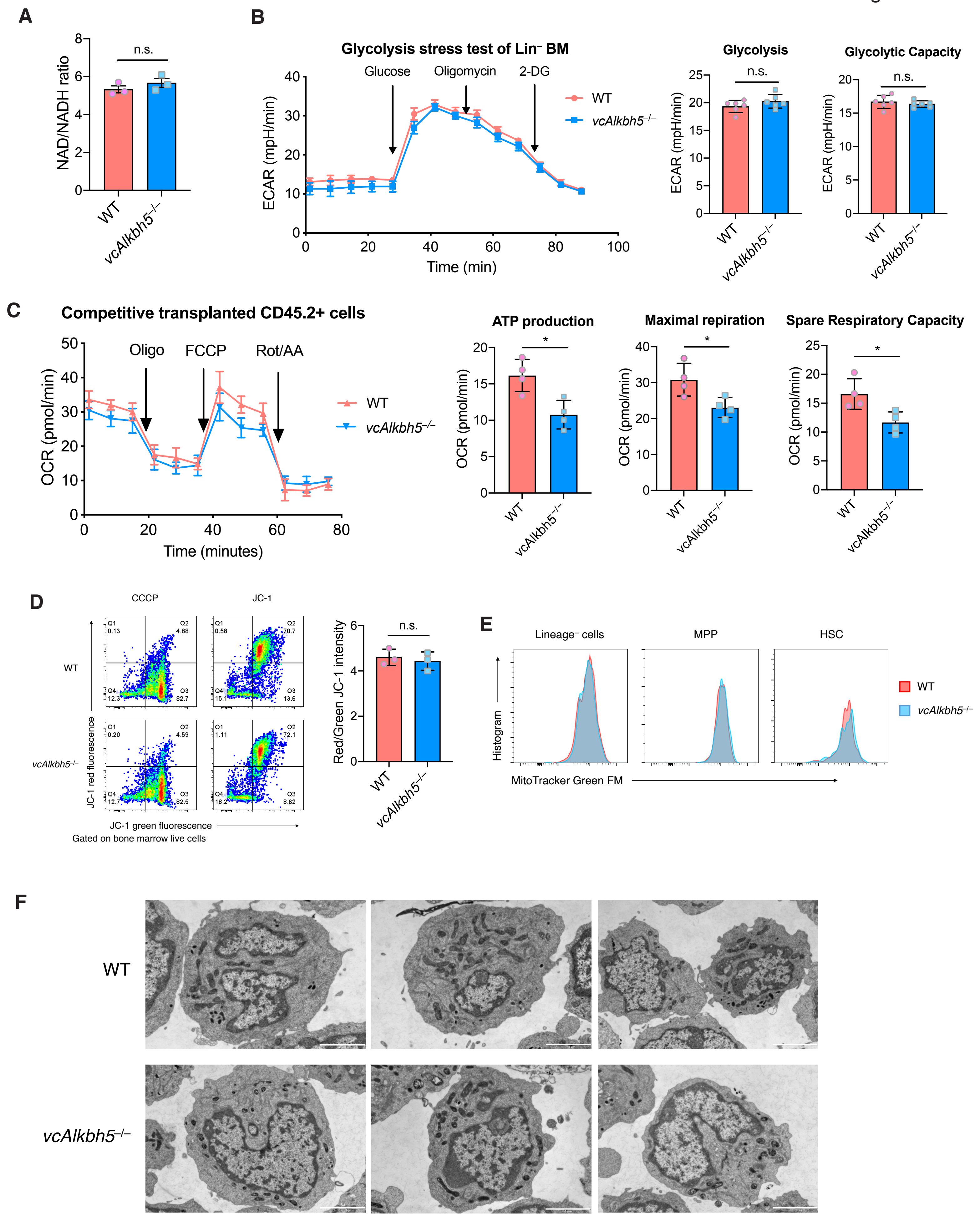
A
n.s.
NAD/NADH ratio
WT
vcAlkbh5–/–
B
Glycolysis stress test of Lin⁻ BM
Glucose
Oligomycin
2-DG
WT
vcAlkbh5–/–
ECAR (mpH/min)
Time (min)
Glycolysis
Glycolytic Capacity
n.s.
C
Competitive transplanted CD45.2+ cells
Oligo
FCCP
Rot/AA
OCR (pmol/min)
Time (minutes)
ATP production
Maximal repiration
Spare Respiratory Capacity
*
D
CCCP
JC-1
JC-1 red fluorescence
JC-1 green fluorescence
Gated on bone marrow live cells
Red/Green JC-1 intensity
E
Lineage⁻ cells
MPP
HSC
Histogram
MitoTracker Green FM
F
WT
vcAlkbh5–/–

Figure S4. Loss of ALKBH5 does not induce apoptosis or damage mitochondrial ultrastructure. Related to Figure 4.

(A) Measurement of NAD+/NADH ratio of lineage-depleted BM cells by ELISA. (B) Determination of glycolysis function via the glycolysis stress test in WT and *vcAlkbh5*$^{-/-}$ lineage-depleted BM cells. (C) Determination of mitochondrial respiration function via measurement of the oxygen consumption rate using the Cell Mito Stress Assay in CD45.2^{+} Lineage-depleted cells of competitive transplanted mice (n = 4 of each group). (D) Mitochondrial health of WT and *vcAlkbh5*$^{-/-}$ hematopoietic cells were determined by MitoProbe™ JC-1 Assay. (E) Characterization of mitochondrial mass change of lineage-depleted cells, MPP and HSC by Mitotracker Green. (F) Ultrastructure of mitochondria in lineage-depleted bone marrow cells of WT and *vcAlkbh5*$^{-/-}$, measured by electron microscope. Scale bar, 2 μm.

Data are represented as mean ± SEM and representative of at least two independent experiments; The p values were calculated using two-tailed Student's t test. n.s. not significant, * $p<0.05$.

Figure S5

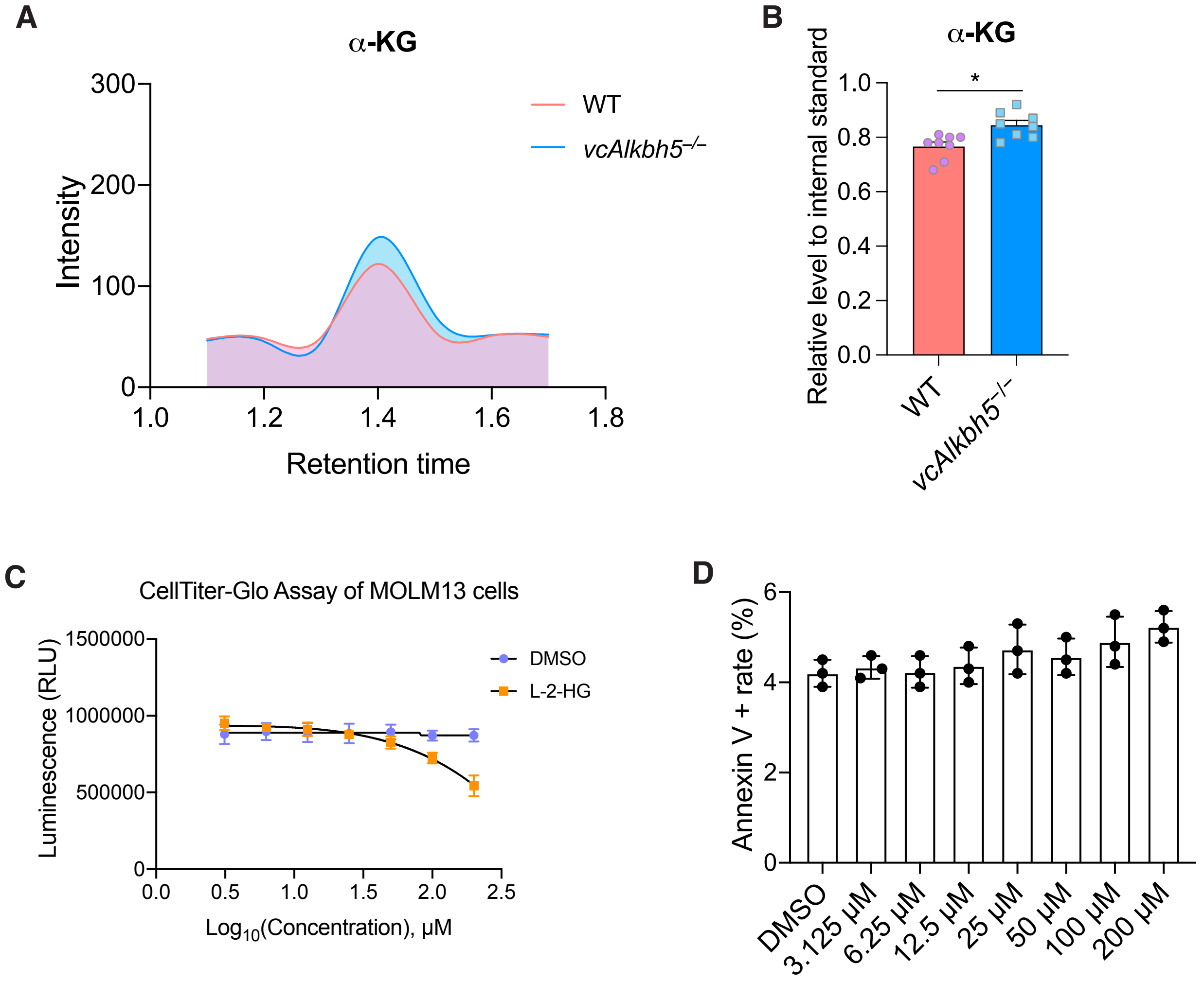
A
α-KG
Intensity
300
200
100
0
1.0
1.2
1.4
1.6
1.8
Retention time
WT
vcAlkbh5−/−
B
α-KG
Relative level to internal standard
1.0
0.8
0.6
0.4
0.2
0.0
*
WT
vcAlkbh5−/−
C
CellTiter-Glo Assay of MOLM13 cells
Luminescence (RLU)
1500000
1000000
500000
0
0.0
0.5
1.0
1.5
2.0
2.5
Log10(Concentration), μM
DMSO
L-2-HG
D
Annexin V + rate (%)
6
4
2
0
DMSO
3.125 μM
6.25 μM
12.5 μM
25 μM
50 μM
100 μM
200 μM

Figure S5. Reduced OGDH results in accumulation of α-KG. Related to Figure 5.
(A) LC–MS analysis of α-KG in the plasma of WT and *vcAlkbh5*$^{-/-}$ mice. (B) Quantification of α-KG levels in murine plasma (n=13). (C) Concentration dependent effect of L-2HG on proliferation of MOLM13 cells. (D) Assessment of apoptosis in response to increasing concentrations of L-2HG assayed by Annexin V staining.
Data are represented as mean ± SEM and representative of at least two independent experiments; The p values were calculated using two-tailed Student's t test. * $p<0.05$.

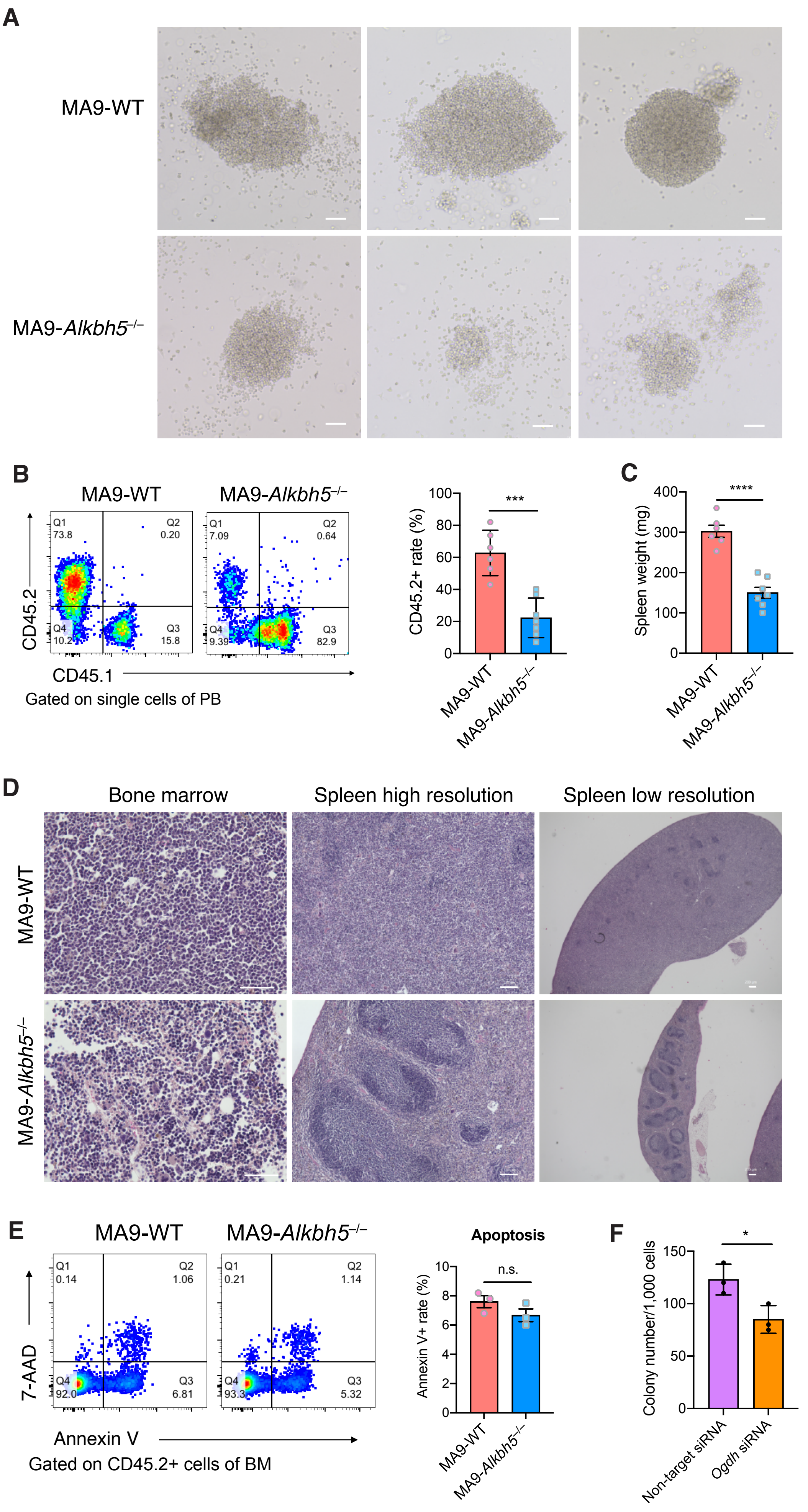
A
MA9-WT
MA9-Alkbh5−/−
B
MA9-WT
MA9-Alkbh5−/−
Q1 73.8
Q2 0.20
Q4 10.2
Q3 15.8
Q1 7.09
Q2 0.64
Q4 9.39
Q3 82.9
CD45.2
CD45.1
Gated on single cells of PB
CD45.2+ rate (%)
***
MA9-WT
MA9-Alkbh5−/−
C
Spleen weight (mg)
****
MA9-WT
MA9-Alkbh5−/−
D
Bone marrow
Spleen high resolution
Spleen low resolution
MA9-WT
MA9-Alkbh5−/−
E
MA9-WT
MA9-Alkbh5−/−
Q1 0.14
Q2 1.06
Q4 92.0
Q3 6.81
Q1 0.21
Q2 1.14
Q4 93.3
Q3 5.32
7-AAD
Annexin V
Gated on CD45.2+ cells of BM
Apoptosis
Annexin V+ rate (%)
n.s.
MA9-WT
MA9-Alkbh5−/−
F
Colony number/1,000 cells
*
Non-target siRNA
Ogdh siRNA

Figure S6. Loss of ALKBH5 diminishes the growth of MA9-*Alkbh5*$^{-/-}$ colonies without increasing apoptotic rate. Related to Figure 6.

(A) Colony morphology of MA9-WT and MA9-*Alkbh5*$^{-/-}$ leukemic cells. Scale bar, 100 μm. (B) Engraftment rate of MA9-WT and MA9-*Alkbh5*$^{-/-}$ leukemia cells (CD45.2^{+}) in congenic CD45.1^{+} recipient mice. (C) Recipient spleen weights at termination of transplantation assay. (D) Histology of bone marrow and spleen of recipient mice transplanted with MA9-WT and MA9-*Alkbh5*$^{-/-}$ cells by H&E staining. Scale bar, 50 μm for the left panel, 100 μm for the middle panel, 200 μm for the right panel. (E) Determination and quantification of MA9-WT *and* MA9-*Alkbh5*$^{-/-}$ leukemia cell apoptotic rate via Annexin V staining (n = 3 of each group). (F) Colony forming unit assay of MA9 cells after transfection with non-targeting or *Ogdh* siRNA.

Data are represented as mean ± SEM and representative of at least two independent experiments; The p values were calculated using two-tailed Student’s t test. n.s. not significant, *** $p<0.001$.
